# Recent advances in bismuth-based heterojunction photocatalysts

**DOI:** 10.3389/fchem.2026.1753678

**Published:** 2026-03-19

**Authors:** Shah Noor, Syeda Maria Hashmi, Muhammad Arif

**Affiliations:** 1 Key Laboratory of Automobile Materials, Department of Materials Science and Engineering, Jilin University, Changchun, China; 2 Institute of Chemical Sciences, Gomal University, D.I.Khan, Khyber Pakhtunkhwa, Pakistan; 3 College of Chemical and Biological Engineering, Shandong University of Science and Technology, Qingdao, China

**Keywords:** bi-based catalysts, crystal structure, H_2_ production, heterojunction catalysts, LBB photocatalysts, sustainable energy

## Abstract

Layered bismuth-based (LBB) nanoparticles, characterized by a unique crystal structure, offer precise control over flaws, band topologies, and morphology, enhancing solar conversion efficiency. Bi’s 6s and O’s 2p orbitals’ hybridization results in a reduced band gap, facilitating improved photo absorption and efficient charge movement. These photocatalysts have proven effective in critical applications, including pollution remediation, CO_2_ reduction, N_2_ fixation, H_2_ production, and O_2_ evolution, positioning them as promising solutions to tackle global environmental challenges. Despite their successes, further research is essential to enhance the photoactivity of LBB photocatalysts to meet stringent industrial criteria for widespread commercialization. Realizing the full commercial application potential necessitates ongoing strides in synthesising, characterising, and modifying bismuth-based photocatalysts. A comprehensive thoughtful of the intricate interplay amid crystal structure and performance is crucial for optimizing their capabilities. As the world shifts its focus toward sustainable and clean energy solutions, bismuth-based photocatalysts emerge as potential major contributors to solving environmental issues and meeting energy needs on a commercial scale. This review highlights the current advancements, trials, and prospects of bismuth-based photocatalysts, emphasizing their pivotal role in fostering a sustainable, cleaner energy future.

## 1 Introduction

**FIGURE 1:**
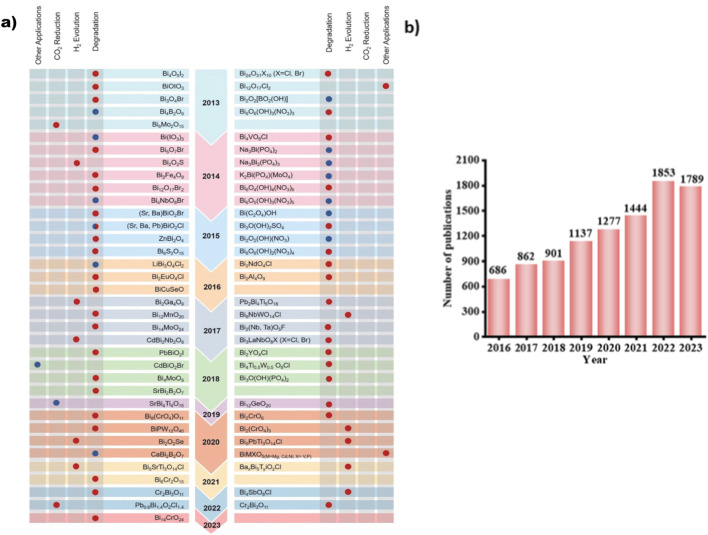
**(a)** The year diagram of the NPBB photocatalysts and their corresponding applications in photocatalysis was first reported (Li et al., 2024) **(b)** The publication count related to 12 distinct types of Layered Bismuth-Based (LBB) materials in conjunction with subjects pertaining to “photocatalysis” since the year 2016. Data adapted from ISI Web of Science, as of 12 January 2023.

Addressing energy shortages and environmental challenges has become critical on a global scale (Yu et al., 2024). The provision of both light and heat energy depends on solar energy, an abundant and clean resource. One exciting new approach to solar energy harvesting is semiconductor photocatalysis, which has widespread uses in organic synthesis, medicine, and other fields (Ahmad et al., 2023; Morshedy et al., 2024; Nosaka and Nosaka, 2017). Since the initial study in 1972, notable advancements have been made in the development of effective photocatalysts (Azhar et al., 2024). Numerous substances have been investigated, such as metallic oxides (TiO_2_, ZnO, Ta_2_O_5_, Fe_2_O_3_), nitrides (Ta_3_N_5_, TaON), sulfides (CdS, MoS_2_), and others (Du et al., 2023; Siedliska, 2024). Materials based on layered bismuth (LBB) have drawn interest due to their hybrid electrical band structure and distinctive layered crystal structure (Kumar et al., 2024; Tao et al., 2024). LBB photocatalysts, including heterojunctions with other semiconductors and bismuth oxyhalides, have been created. Bi 6s and O 2p orbital hybridization widen the hybridized state, producing dispersive band structures for quick charge movement and a thin band gap for better photo absorption (Lv et al., 2022; Shi et al., 2023). Although LBB photocatalysts have the potential to be used in applications such as H_2_ generation and pollution purification, their photoactivity is not up to industrial standards (Bamiduro and Zahran, 2025; Tian et al., 2022). Numerous approaches are being investigated for improving light absorption and active site abundance to increase photocatalytic activity (Cheng et al., 2014; Gao et al., 2024; Henrotte et al., 2025; Huang et al., 2014). Because of these remarkable qualities, LBB photocatalysts have attracted much attention lately, as evidenced by the noteworthy rise in publications over the last 10 years (Hassaan et al., 2023; Mohamadpour and Mohammad Amani, 2024). They still need to increase their photoactivity; thus, it is time to analyze LBB photocatalysts thoroughly. Thoughtful reviews of bismuth-based photocatalysts are available, but a thorough examination of the structural traits, photocatalytic properties, and systematic classification of the full range of LBB materials is lacking (Cao et al., 2014; Bamiduro and Zahran, 2025; Morshedy et al., 2024). The correlation between performance and crystal structure has received relatively less attention in the reviews that have already been published (Lv et al., 2022; Shi et al., 2023). Figure 1 provides an overview of bismuth-based photocatalysts and their potential applications from 2013 to 2023.

This paper attempts to give a thorough review of the latest developments in LBB photocatalysts, including their classifications, structural properties, synthesis, characterization, and applications, as well as a synopsis of their possible uses. As we explore avenues for a sustainable future, we hope to contribute to the design of operative Bismuth-based photocatalysts for critical processes (Tian et al., 2022).

## 2 A brief overview of photocatalysts and their potential applications

**FIGURE 2:**
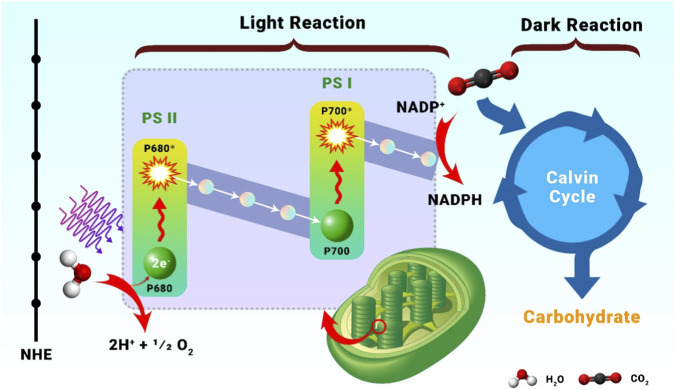
The Z-scheme elucidates the charge separation processes occurring in nature during photosynthesis, specifically in Photosystems I and II, employing protein photocatalysts. P680 and P700 are denoted as P680* and P700*, respectively, for the excited states of chlorophyll. The intricate sequence involves the generation of high-energy electrons and their transfer between photosystems, culminating in the reduction of NADP (nicotinamide adenine dinucleotide phosphate reductase). From the Ref (Ishikita et al., 2006).

Photocatalysis is inspired by natural photosynthesis, in which essential light-driven processes conform to the “Z-scheme,” including PSI and PSII photosystems, among other vital components (Figure 2). In PSII, photons (P680) are absorbed by chlorophylls (Lubitz et al., 2006), which then uses that energy to remove electrons from H_2_O and produce O_2_ by the action of water oxidation catalysts (WOCs). After being separated, these electrons are sent to PSI, where photons (P700) are absorbed by chlorophylls, further exciting the electrons and promoting reduction reactions like NADP^+^ to NADPH by the Proton gradient-driven processes, such as this power downstream transformations, and the Calvin cycle’s conversion of CO_2_ to hydrocarbons. Optical energy is efficiently used by natural photosynthesis to propel chemical reactions (Huang et al., 2014).

**FIGURE 3:**
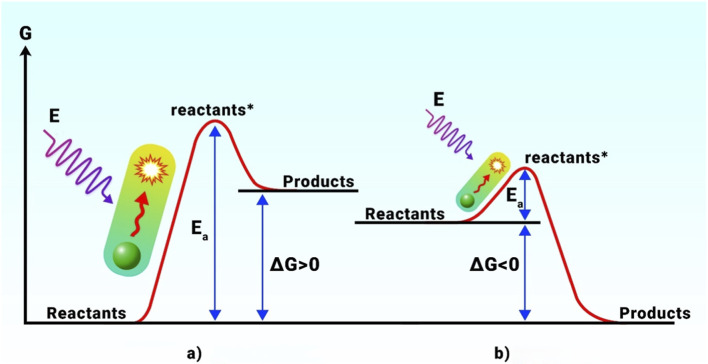
Energy dynamics of non-spontaneous and spontaneous photocatalytic reactions: **(a)** uphill procedure; **(b)** downhill procedure. From the Ref (Mohamadpour and Mohammad Amani, 2024; Navidpour et al., 2023).

The idea of photocatalysis dates to Edmond Becquerel in 1839, and pioneers such as Boddy Honda and Fujishima helped it acquire significant traction in the late 1960s. Current photocatalysis research aligns with sustainability objectives; one possible use is the direct chemical storage of solar energy, sometimes known as artificial photosynthesis or solar fuels. Two separate processes are involved in photocatalysis (Figure 3): promoting photosynthesis through thermodynamically uphill (ΔG > 0) reactions and facilitating thermodynamically downhill (ΔG < 0) reactions by altering reaction kinetics (Yudin, 2020). IUPAC defined “photocatalyst” as any catalyst that can trigger chemical processes when light is absorbed.

Our comprehensive review’s main goal is to provide researchers with a comparative framework covering a variety of topics, such as general benefits and drawbacks, charge separation fundamentals, optimization techniques, electronic structure issues, synthesis methods, oxyhalide heterojunctions, and various bismuth-based heterojunction photocatalytic materials. The discussion goes on to distinguish distinct types of photocatalysts according to how they promote particular chemical changes, such as the splitting of water (Kumar et al., 2024), the reduction of CO_2_, the breakdown of pollutants, and different types of organic processes (Nogueira et al., 2022). The review provides insights into prospective directions for future research and development in the discipline, further outlining potential applications.

## 3 Introduction to bismuth-based photocatalysts

Photocatalysts containing bismuth have garnered significant consideration in current ages because of their irreplaceable characteristics and potential uses in photocatalysis (Arif et al., 2021; Shah et al., 2023). Bismuth, a heavy metal with a diverse range of oxidation states, introduces distinctive electronic structures that contribute to the photocatalytic functioning of these materials. Bismuth-based photocatalysts are being explored for several applications, including pollutant degradation, hydrogen evolution, and polymerase chain reaction (PCR) enhancement (Shi et al., 2023). The electronic properties of bismuth enable the manipulation of conduction band positions, bandgap engineering, and the creation of defects, all of which play crucial roles in photocatalytic processes. The framework and development of bismuth-based photocatalysts involve oxygen vacancies, bismuth-rich compositions, and incorporating other elements to enhance their photocatalytic efficiency (Navidpour et al., 2023; Walter et al., 2010; Yudin, 2020). These materials hold promise for addressing environmental challenges and advancing technologies that rely on photocatalytic processes. Exploring bismuth-based photocatalysts is an evolving and dynamic field in materials science and environmental research.

### 3.1 Types of bismuth-based photocatalysts

The performance of photocatalysis is intricately linked to the crystal lattice and electronic configuration. This section analyses the electrical and crystalline structures of well-known bi-based photocatalysts and how they affect photocatalytic activity. This analysis is of paramount significance, offering insights to steer and refine strategies to acquire highly efficient photocatalysts.

#### 3.1.1 Bi_2_O_3_ and Bi_2_S_3_

**FIGURE 4:**
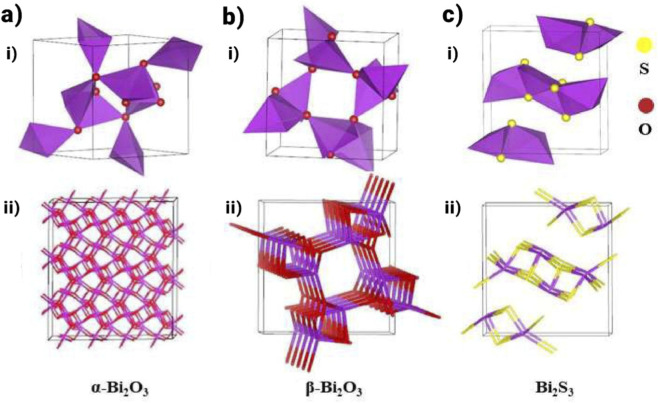
**(a,b)** The crystal structure model of Bi_2_O_3_ (Cheng et al., 2010), **(c)** The crystal structure model of Bi_2_S_3_ (Wang Y. et al., 2009).

Figures 4a,b displayed the frameworks of distinct crystalline phases of Bi_2_O_3_. The α-phase (room temperature) and the δ-phase (high temperature) are both constant phases. In contrast, the β-Bi_2_O_3_’s photocatalytic performance surpasses that of α-Bi_2_O_3_, primarily because β-Bi_2_O_3_ has a narrower band gap and greater absorption of visible light (Janardhana et al., 2025; Mane et al., 2024). Figure 4c displays that the crystal structure model of Bi_2_S_3_ was observed from the c-axis. The narrow band gap of Bi_2_O_3_ has been reported to face the challenge of rapid recombination of photogenerated electron-hole pairs. Bi_2_O_3_ exhibits notable absorption within the visible spectrum, having a band gap between 2.1 and 2.8 eV. Bi_2_O_3_ is divided into four phases: monoclinic (α-Bi_2_O_3_), body-centered cubic (γ-Bi_2_O_3_), tetragonal (β- Bi_2_O_3_), and face-centered cubic phase (δ-Bi_2_O_3_). Significantly, β-Bi_2_O_3_ has demonstrated superior photocatalytic performance compared to α-Bi_2_O_3_, attributed to its unique structural characteristics that facilitate channels for efficient charge carrier transfer, thereby mitigating recombination during the photocatalytic process. Despite the advantageous properties of β-Bi_2_O_3_, its metastable state poses a challenge in developing facile methods for the preparation of pure β-Bi_2_O_3_, particularly on the nanoscale (Wang et al., 2013). Current research efforts towards Bi_2_O_3_ primarily focus on enhancing the photocatalytic performance of α-Bi_2_O_3_ (Hezam et al., 2018; Wang et al., 2013). Bi_2_S_3_, a widely studied semiconductor in the V-VI group, is a direct n-type semiconductor with a narrow band gap ranging from 1.3 to 1.7 eV. Within the orthorhombic crystal system, its molecules, with differing bond lengths, are anisotropically connected through Bi-S bonds and form a regular layered structure. As a result, macroscopic formations of various shapes can develop.

Bi_2_S_3_, characterized by a compressed band gap ranging from 1.3 to 1.7 eV, readily generates photogenerated carriers upon exposure to visible and near-infrared light, making it a widely employed stability sensitizer. Typically existing in an orthorhombic phase with a layered structure, Bi_2_S_3_ encounters challenges in photocatalytic applications due to the fast reuniting of electrons and holes created by light when used in isolation. Consequently, with other materials to form heterojunctions, numerous studies have investigated the coupling of Bi_2_S_3_ to address this limitation and enhance overall photocatalytic efficiency (Cheng et al., 2011).

#### 3.1.2 BiVO_4_

**FIGURE 5:**
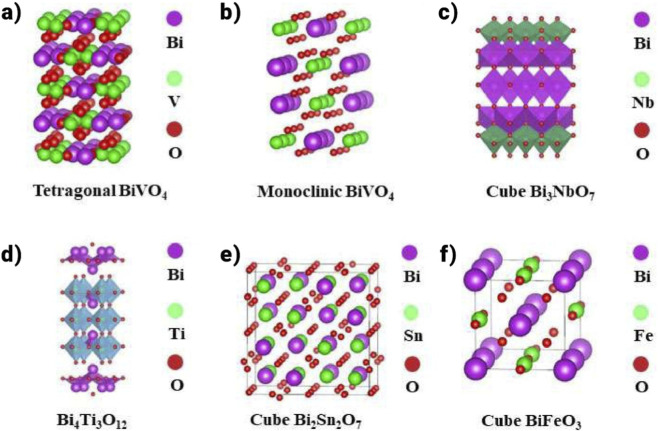
**(a,b)** Crystal structure model of BiVO_4_, **(c)** Crystal structure model of Bi_3_NbO_7_ (Zhang G. et al., 2009), **(d)** Crystal structure model of Bi_4_Ti_3_O_12_ (Yao et al., 2003), **(e)** Crystal structure model of Bi_2_Sn_2_O_7_, **(f)** Crystal structure model of BiFeO_3_.

Tetragonal zircon structures (t-zBiVO_4_), monoclinic scheelite (mBiVO_4_), and tetragonal scheelite (tBiVO_4_) are the three observed polymorphs of BiVO_4_. One of these is t-zBiVO_4_, which has a 2.9 eV band gap and exhibits comparatively low photocatalytic activity when exposed to visible light. Much research has been done on the scheelite form of BiVO_4_ photocatalyst, which has a band gap of 2.4 eV (Figures 5a,b). The conduction band (CB) minimum and valence band (VB) maximum in mBiVO_4_ and tBiVO_4_ are located in the same places, indicating that the thermodynamic limitations on photocatalytic reactions are similar (Tokunaga et al., 2001).

On the other hand, mBiVO_4_ demonstrates better photocatalytic activity, highlighting the significant impact of charge kinetics, in which charge carrier transport predominates over photogenerated electron-hole participation in the reaction. Variations in an alteration of a confined environment account for the apparent difference in the photocatalytic reaction rate amongst the dual forms of BiVO_4_. A 6s^2^ lone pair of Bi^3+^ in mBiVO_4_ causes a more significant deformation of the Bi–O polyhedron than in tBiVO_4_, leading to effective charge transfer. DFT studies based on electronic structure predictions show that O 2p and Bi 6s orbitals dominate the VB of mBiVO_4_, whereas V 3d orbitals principally control the CB (Zhao et al., 2011). The configuration and alignment of particles in semiconductor photocatalysts greatly impact their catalytic efficacy, which is explained by the impact of atomic coordination and arrangement on various surfaces concerning CO_2_ adsorption, activation, and photogenerated current (Zhang G. et al., 2021).

One important aspect is the movement of bismuth atoms, and a useful tactic to improve the photocatalytic activity of bismuth-based materials is to modify the crystal planes. In a final experiment, controlled cobalt doping was used to insert Co_3_O_4_ on the (110) plane of BiVO_4_. The effective hole capture on the crystal plane by the doped Co_3_O_4_ is used to achieve the excellent parting of photogenerated electron-hole pairs. As shown in Figure 5d, Bi_4_Ti_3_O_12_ is a layered wide band gap semiconductor comprising three layers of TiO_6_ octahedra and one layer of [Bi_2_O_2_]^2+^ alternately stacked along the c-axis (Cu et al., 2016). Ti 3d and Bi 6p orbitals are said to make up the majority of the conduction band (CB) of Bi_4_Ti_3_O_12_, whereas O 2p and Bi 6s hybrid orbitals are the source of the valence band (VB) (Cu et al., 2016). The photocatalytic performance of bismuth titanate is influenced by the production method and molar ratio of Bi/Ti due to the active groups in TiO_6_ octahedra and TiO_4_ tetrahedra. However, limited carbon dioxide adsorption performance and delayed charge kinetics make using a single bismuth titanate as a photocatalyst impractical. Liu et al. modified magnetic domain orientation to increase active sites and decrease recombination of photogenerated electron-hole pairs. Bi_4_Ti_3_O_12_ improved photocatalytic performance due to increased surface charge transfers and better CO_2_ adsorption. As a photocatalyst, Bi3NbO7 (BNO) has good chemical stability and visible light responsiveness, making it a promising candidate for aqueous environment purification. Nevertheless, the photocatalytic activity is significantly limited by the high rate of photogenerated carrier complexation. In this work, S-scheme heterojunction composite photocatalysts were prepared by adding urea using the one-pot solvent approach, which increased the photocatalytic capacity (Figure 5c).

#### 3.1.3 Bismuth stannate (Bi_2_Sn_2_O_7_)

The p-type semiconductor Bi_2_Sn_2_O_7_ (Figure 5e), which has a band gap ranging from 2.3 to 2.8 eV, exhibits magnetic properties and exists in three distinct crystal structures: α (monoclinic), β (face-centered cubic), and γ (cubic). Its lattice structure changes depending on the temperature at which it is calcined (Shannon et al., 1980). The lone pair of bismuth, generated by the hybridization of the 6s and 6p orbitals, reduces the spatial symmetry in its oxidized state, resulting in a distorted pyrochlore structure. In the Bi_2_Sn_2_O_7_ pyrochlore, the valence band (VB) is primarily composed of O 2p and Bi 6s orbitals, while the conduction band (CB) consists of O 2p, Sn 5s, and Bi 6p orbitals. Bi_2_Sn_2_O_7_ is considered a promising material for use as a semiconductor photocatalyst when its band gap is within the appropriate range. One common method for synthesizing this material is the high-temperature solid-phase reaction technique (Zhao et al., 2017) and the thermal decomposition method (Gnanamoorthy et al., 2018). Because of its low photocatalytic activity, pure Bi_2_Sn_2_O_7_ is not frequently used despite its potential. Scholars have endeavored to augment electron-hole pair separation using diverse methodologies, such as establishing heterojunctions to attain an ideal band gap for amplified charge transfer effectiveness (Zhou et al., 2022). By absorbing electrons and producing CO^2−^, oxygen vacancies (OVs) and metal coordination unsaturated spots are recognized as contributors to CO_2_ reduction, augmenting the photocatalytic reduction process (Xi et al., 2012). For example, reducing the size of Bi_2_Sn_2_O_7_ nanoparticles can ease the insertion of oxygen vacancies (Guo et al., 2020). 8.1 times more CO_2_ was converted to CO by Bi_2_Sn_2_O_7_ nanoparticles than by untreated Bi_2_Sn_2_O_7_ nanoparticles. Furthermore, DFT calculations show that oxygen vacancy introduction can lower the CO desorption step’s energy barrier.

#### 3.1.4 BiFeO_3_

BiFeO_3_, with a narrow band gap of about 2.0–2.8 eV, is an attractive semiconductor material due to its excellent properties, such as low cost, non-toxicity, chemical stability, and magnetic characteristics (Marwat et al., 2022). In the crystal structure of BiFeO_3_, the A site is occupied by cations with a larger ionic radius, which are coordinated with 12 oxygen atoms. In comparison, cations with a smaller ionic radius occupy the B site and are coordinated with 6 oxygen atoms. This structure follows the typical ABO_3_ perovskite arrangement, where oxygen anions are positioned at the center of the unit cell faces, the Bi cations (A) are located at the corners, and the Fe cations (B) sit in the center of the unit cell. In Figure 5f, the crystal structure model of Bismuth Ferrite (BiFeO_3_) is shown. The valence band (VB) is formed by the hybridization of the O 2p state and the Fe 3d orbital, while the conduction band (CB) consists of the O 2p and Bi 6p states. The interaction between the O, Fe, and Bi orbitals creates a band gap calculated to be 2.8 eV using the DFT-based shield exchange method. However, predictions suggest that BiFeO_3_ may have an indirect band gap smaller than the direct band gap, estimated between 0.4 and 1.0 eV (Wang H. et al., 2009; Wang et al., 2012). The sol-gel method has been frequently employed for the preparation of BiFeO_3_ (Haruna et al., 2020). Bismuth ferrite has recently garnered significant attention due to its distinctive magnetic and photocatalytic properties. The formation of heterojunctions in BiFeO_3_ composites has proven effective in reducing electron-hole recombination, enhancing the separation of photogenerated electron-hole pairs, and significantly boosting catalytic efficiency. For example, Amit Kumar et al. designed an Ag_2_CrO_4_/Ag/Bi-FeO_3_/rGO Z-type catalyst that effectively separated photogenerated carriers. Remarkably, this catalyst achieved a CO_2_ reduction rate to CH_4_ of 30.25 μmol⋅g^-1^h^-1^. The outstanding photocatalytic performance is attributed to the formation of a Z-type heterojunction at the interface, which promotes efficient carrier separation and expands the photoresponsivity range. Bi_2_MO_6_ (M = Mo, W).

The Bi_2_MO_6_ (M = Mo, W) system has two crystallographic phases: orthorhombic and monoclinic structures. The monoclinic structure is a high-temperature phase (T > 960 °C), whereas the orthorhombic structure predominates at low temperatures (T > 960 °C). The orthorhombic structure of the Bi_2_MO_6_ (M = Mo, W) photocatalysts is the main subject of current research. These compounds are the most basic members of the oxide family of Aurivillius type, with [MO_6_] octahedral layers sandwiched between [Bi_2_O_2_]^2+^ layers (Arif et al., 2021; Sun and Wang, 2014).

**FIGURE 6:**
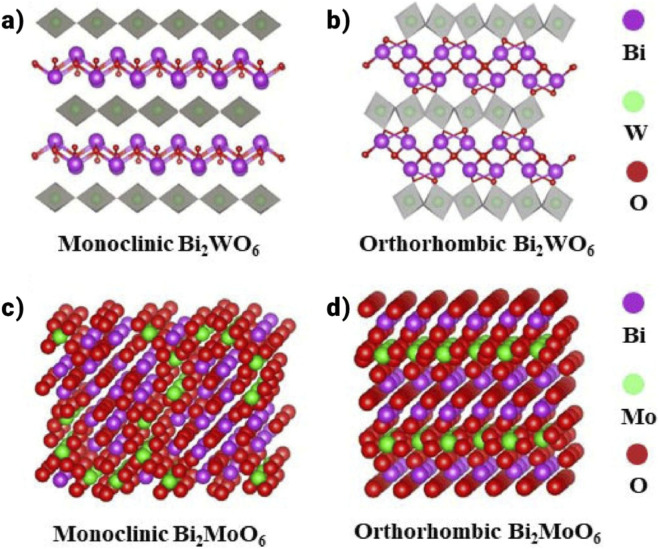
**(a)** Schematic representation of the Bi_2_WO_6_ crystal structure, **(b)** Depiction of the Bi_2_WO_6_ crystal lattice model, **(c)** Model of the Bi_2_MoO_6_ crystal structure, **(d)** Illustration of the Bi_2_MoO_6_ crystal framework (Di et al., 2019a).

Distortions are observed in the local structures of M and Bi ions in orthorhombic Bi_2_MO_6_ (Figures 6a,b) (M = Mo, W). Theoretical calculations reveal that the valence band (VB) of Bi_2_MO_6_ (M = Mo, W) is primarily composed of O 2p and Bi 6s orbitals, while the conduction band (CB) is dominated by X nd orbitals (Mo 4d, W d). The band gaps of Bi_2_MoO_6_ (Figures 6c,d) and Bi_2_WO_6_ are approximately 2.6 eV and 2.8 eV, respectively (Li et al., 2015).

#### 3.1.5 BiOX (X = Cl, Br, I)

BiOX (X = Cl, Br, I) usually crystallizes in the tetragonal matlockite structure, which is characterized by two layers of X− interspersed with layer formations of [Bi_2_O_2_]^2+^ along the direction of the z-axis. The polarization of linked atoms and orbitals can occur freely in this open-layer crystalline structure, producing internal static electric fields perpendicular to the [Bi_2_O_2_]^2+^ and X− layers (Ding et al., 2017). The efficient separation of photogenerated electron-hole pairs is enhanced by the formation of an internal electric field between the layers, which greatly boosts the photocatalytic performance of BiOX (X = Cl, Br, I). The electronic structure of BiOX is primarily composed of Bi 6p orbitals in the conduction band (CB), while the valence band (VB) is dominated by O 2p and X np orbitals (n = 3, 4, and 5 for X = Cl, Br, and I, respectively). Interestingly, the contribution of X ns orbitals increases with the atomic number of Cl, Br, and I, resulting in a more pronounced dispersion of band energy levels. Consequently, the band gap decreases from BiOCl to BiOBr and BiOI (3.2 eV, 2.7 eV, 1.7 eV, respectively (Cheng et al., 2014; Ding et al., 2017).

#### 3.1.6 Bi_2_O_2_CO_3_

In particular, (Bi_2_O_2_CO_3_) (BOC) has garnered a lot of attention lately for the electrochemical reduction of CO_2_-to-formates because the Bi–O bond helps to improve the reaction kinetics and selectivity of CO_2_ in orthogonal symbiosis between [Bi_2_O_2_]^2+^ and [CO_3_]^2-^ layer, which provides unique electronic structures and highly asymmetric intrinsic structure for modified catalytic properties. Fortunately, Bi_2_O_2_CO_3_ may be easily produced by reconstructing Bi-based precursors, such as Bi-MOF, BiPO_4_, and Bi, which undergo conversion and dissociation mediated by surrounding reactants and products under working conditions. Nevertheless, the potential-mediated rebuilding will produce metallic Bi^0^ with disordered morphologies, such as Bi nanoparticles, nanosheets, and nanoleaves, making chemical valence states and active sites uncontrollable. Restricting the step rebuilding of Bi_2_O_2_CO_3_ toward increased activity is therefore very desirable (Zhang et al., 2023).

#### 3.1.7 Bi_8_(CrO_4_)O_11_

Kumada et al. initially investigated the single crystal Bi_8_(CrO_4_)O_11_ with a layered structure; they did not investigate its photocatalytic activity. Up to 2020, Zhu’s group successfully synthesized and employed Bi_8_(CrO_4_)O_11_, a wide-spectrum (about 678 nm) responsive catalyst, for the photodegradation of phenol. Compared to CdS and P25-TiO_2_, phenol’s photodegradation activity over Bi8(CrO_4_)O_11_ is approximately 23.0 and 2.9 times higher, respectively. Despite having an acceptable energy band structure and a moderately favorable valence band potential, Bi8(CrO_4_)O_11_ still has several issues that need to be resolved, like limited electronic conductivity and a high rate of photogenerated electron-hole pair recombination. G-C_3_N_4_, a reduction photocatalyst, has a more negative conduction band position, whereas Bi8(CrO_4_)O_11_, an oxidation photocatalyst, has a bigger work function and a more positive valence band (Gu et al., 2022). Comparison of different types of Bi-based materials towards pollutants removal and their performance metrics is given in Table 1.

**TABLE 1:** Single-component bismuth photocatalysts.

Catalyst	Target reaction	Performance metric	Value(s)	QE/Proxy	Stability (cycles/% retained)	Ref.
Bi_2_O_3_ (β-phase)	MB degradation (vis)	Degradation/k	100%/∼6 h; k = 0.012 min^-1^	AQE 2.1% @450 nm	4/87%	Cadenbach et al. (2025), Oladipo and Mustafa (2023)
BiVO_4_ (bare)	PEC water oxidation	Photocurrent	1.0 mA/cm^2^ @1.23 V	τ = 15 ns	3/85%	Long et al. (2024), Wakjira et al. (2024)
Bi_2_MoO_6_ (M = Mo)	TC degradation	k/Degradation	0.0186 min^-1^; 88%/75 min	AQE 3.8% @460 nm	5/92%	Feng et al. (2023), Li et al. (2025)
BiOX (BiOBr, microsphere)	Dye degradation	Degradation rate	92% RhB/120 min	AQE 4.2% @470 nm	5/89%	Long et al. (2024), Wakjira et al. (2024)
Bi_2_O_2_CO_3_	MB degradation (vis)	k	12 × 10^−2^ h^-1^ (0.002 min^-1^)	AQE 1.9% @420 nm	4/88%	Oladipo and Mustafa (2023)

### 3.2 Synthesis and characterization techniques for bismuth-based photocatalysts

Various analytical techniques, including XRD, BET, SEM, EDS, and FT-IR, were employed to characterize the microstructure and properties of bismuth-based photocatalysts. In particular, the D/Max2550VB X-ray diffractometer can be used to analyze the phase composition of bismuth-based photocatalysts. The N22-27E pore analyzer and high-speed automatic surface area were employed to determine the specific surface area and pore size of the bismuth-based photocatalysts. The degassing procedure was conducted at 100 °C using nitrogen as the adsorbate. It is well established that the synthesis techniques significantly influence the size, shape, and specific surface areas of photocatalysts, which in turn greatly affect their adsorption properties and photocatalytic activity. Additionally, these synthesis routes have implications for environmental impact, synthesis scale, production cost, and safety considerations (Cheng et al., 2014; Reverberi et al., 2018). The synthesis of Bi-based photocatalysts primarily involves Bi sources such as Bi(NO_3_)_3_.5H_2_O, NaBiO_3_.2H_2_O, BiCl_3_, Bi_2_O_3_, and elemental Bi.

#### 3.2.1 Hydrothermal/solvothermal method

**FIGURE 7:**
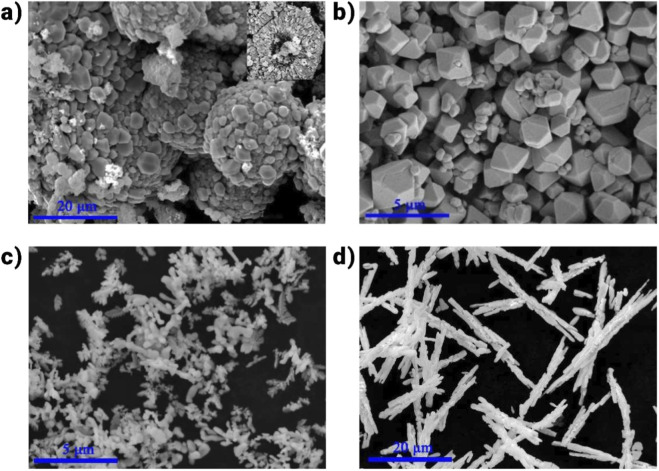
SEM images of BiVO_4_ powders produced at pH= **(a)** 0.5, **(b)** 2, **(c)** 7, and **(d)** 12 (Lin et al., 2019).

One of the main techniques for creating Bi-based photocatalysts is the hydrothermal/solvothermal process, which provides control over facets, size, surface imperfections, morphology, and dimensionality. Precise control over these parameters is possible through modifications to the pH level, solvent, reaction duration, and temperature. When it comes to photocatalysts, the hydrothermal/solvothermal process typically yields higher-quality nanoparticles that are better suited for specific uses than dry methods (Reverberi et al., 2018). The low production rate of this approach is a noteworthy downside, which is linked to the longer production time and batch-oriented nature induced by the usage of specialist autoclaves. Risks associated with the hydrothermal/solvothermal approach include the possibility of toxic solvent emissions and the leakage of nanoparticles into the water (Stieberova et al., 2019). Lin and colleagues have synthesized mBiVO_4_ photocatalysts successfully using the hydrothermal technique (Lin et al., 2019). The morphologies and sizes of mBiVO_4_ produced under varying pH settings were seen to alter, as illustrated in Figures 7a–d. Notably, the microstructure and photocatalytic activity of BiVO_4_ was significantly influenced by the pH level. Coralloid particles synthesized at pH = 7 exhibited outstanding photocatalytic degradation capability for Rhodamine B (RhB) under visible light irradiation. This superior photocatalytic performance can be attributed to their enhanced capacity for charge carrier separation and efficient solar energy absorption.

Additionally, it has been investigated how the pH value plays a crucial role in controlling facet exposure, which has an impact on photocatalytic performance (Hassaan et al., 2023). For example, BiVO_4_, which the Colon group researched, clearly changes morphologically with pH from a ball shape to a needle-like morphology. In a related study, Sarkar et al. used several solvents and surfactants to create Bi_2_S_3_ nanoparticles with a range of sizes and forms using the solvothermal approach (Sarkar et al., 2016). Due to their huge specific surface areas, the Bi_2_S_3_ nanoparticles produced from a mixture of trioctylphosphine oxide and oleylamine showed the strongest photocatalytic performance. Additionally, reducing solvents can be used purposefully to create surface flaws in Bi-based photocatalysts. For instance, olive-green, few-layered BiOI with enhanced (001) facets and increased spacing of oxygen vacancies was successfully synthesized using the hydrothermal method, with ethylene glycol serving as the solvent (Ye et al., 2016a). It was discovered that this material modification improved CO_2_ photoreduction.

#### 3.2.2 Solid reaction method

When creating Bi-based photocatalysts, the hydrothermal/solvothermal approach has proven to have many benefits, most notably in producing a tunable nanostructure. However, a substantial volume of water is required if commercial Bi-based photocatalysts are made using this technique. On the other hand, the large-scale, production-friendly solid reaction approach sticks out as a viable substitute that does away with the expensive organic solvents and water that are usually utilized in solvothermal procedures. However, it is crucial to remember that the solid reaction process is less environmentally friendly because there is a greater chance that it will release nanoparticles into the air (Gottschalk and Nowack, 2011). Due to the more challenging regulation of the production process in solid reaction synthesis compared to wet methods, the resulting product often exhibits a broader size distribution (Bai et al., 2018). For instance, Bi_3_O_4_ClxBr_1−x_ is synthesized through the calcination of Bi_2_O_3_ and BiOX (Cl, Br) at 400 °C (Cuéllar et al., 2011). This can be attributed to the relatively weak Van der Waals interactions of the halogen atoms, which allow for easy substitution in bismuth oxyhalide. Additionally, Bi_2_O_3_ and MoO_3_ are calcined at 550 °C to create γ-Bi_2_MoO_6_ powder (Cuéllar et al., 2011; Zhang et al., 2025). From the γ-Bi_2_MoO_6_ powder, a sequential process of breakdown and evaporation is then used to generate a γ-Bi_2_MoO_6_ film with improved photocatalytic performance.

#### 3.2.3 Template method

The template method is particularly noteworthy among the various synthesis techniques, offering a reliable approach for the controlled production of Bi-based photocatalysts. This method enables the formation of highly ordered multidimensional shapes or hollow structures, which are challenging to achieve through direct synthesis methods (Liu et al., 2013). The template method can be further categorized into hard template, soft template, and self-template approaches, depending on the types of templates employed. However, this method is associated with time-consuming processes and high costs related to both template synthesis and removal. While SiO_2_ templates are inexpensive, simple to manufacture, and change, there are concerns regarding their environmental impact because removing them frequently necessitates the use of extremely corrosive acids or bases (Joo et al., 2013). Furthermore, rather than taking economic implications into account, a large portion of the research that has already been done favors templates based on their functional characteristics. Specifically, the self-template method eliminates the need for extra templates, providing a more practical and cost-effective solution for real-world applications. As a result, synthesis procedures are streamlined, and production costs have drastically decreased (Joo et al., 2013). For instance, Xiao et al. presented a self-template technique for creating a hollow hierarchical structure of Bi_2_WO_6_ that resembles a rod (Xiao et al., 2018). In this self-template technique, Bi precursor micro rods are produced by rapid hydrolysis of Bi(NO_3_)_3_ in water, and they act as sacrificial templates.

The reaction graph for the hydrolysis is as follows:

$$6Bi{\left(NO_{3}\right)}_{3}+11H_{2}O\;\to \;{Bi}_{6}O_{5}{\left(OH\right)}_{3}{\left({NO}_{3}\right)}_{5}.{3H}_{2}O+13HNO_{3}$$ (2)

**FIGURE 8:**
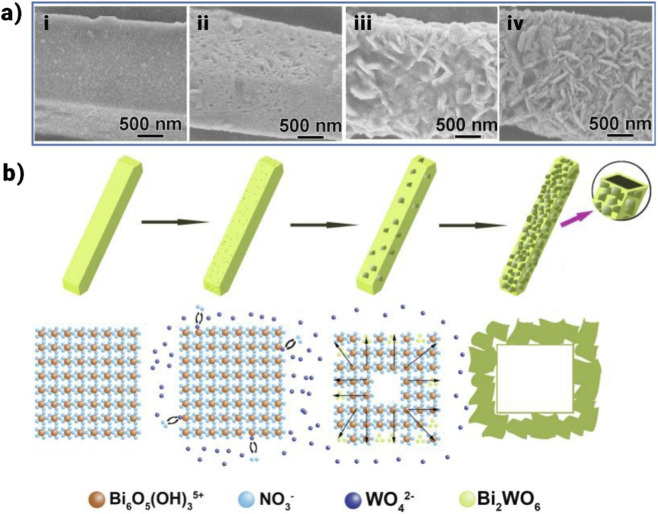
**(a)** SEM pictures depict the intermediary products generated throughout the production of Bi_2_WO_6_ Hollow Hierarchical Rods (HHRs) at various reaction times (i-iv: 0, 5, 10, and 20 h), **(b)** schematic design elucidates the evolutionary process, delineating the transformation from Bi precursor microrods to the final Bi_2_WO_6_ HHRs (Xiao et al., 2018).

Using the Kirkendall phenomenon, microrods can be transformed into hollow hierarchical structures, as seen in Figure 8. An anion exchange mechanism first exchanges WO_4_^2–^ anions with NO^3–^ anions inside the Bi precursor microrods. Then, in a hydrothermal reaction with WO_4_^2–^ anions, the Bi_6_O_5_ (OH)_3_^5+^ polycations produce Bi_2_WO_6_. Consequently, the created Bi_2_WO_6_ nuclei of the surface originally serve as nucleation sites, allowing later-formed Bi_2_WO_6_ species to diffuse to the surface. In the end, the mass transfer mismatch leads to the creation of Bi_2_WO_6_ microrods.

#### 3.2.4 Precursor method

Because different precursors have different melting temperatures, solubility, and stability, using them can produce different reactions. Bi-based photocatalysts can have customized morphologies by using bismuth sources as precursors. It was noted in a study by Florez-Rios et al. (2020) that bismuth acetate and bismuth subsalicylate can be used as Bi precursors to produce the crystal structure of BiOI. The bismuth acetate-derived BiOI had a morphology resembling a flower, while the bismuth subsalicylate-derived BiOI had a structure resembling a dandelion with comparable microstructures. A typical tactic to improve photocatalytic performance in the precursor technique of bismuth-based photocatalyst synthesis is to modify the photocatalyst’s exposed crystal face by altering pH. It has been claimed that controlling the pH of the solution allows for control over the exposed crystal facets of BiOI. More species with an exposed (001) face of BiOI are formed when the pH is kept below 7, whereas BiOI with the face becomes the predominant crystal face at pH values higher than 7 (Liu et al., 2023; Liu et al., 2025).

#### 3.2.5 Solid-phase reaction method

Under specific thermodynamic conditions, two or more solids are combined through the solid-phase reaction method to form solid compounds or powders (Ikesue et al., 1995). Large-scale manufacturing is better suited for the solid-phase reaction approach than the hydrothermal method (Dolla et al., 2025; Minkiewicz et al., 2023). However, it has the disadvantage of perhaps causing more airborne nanoparticle releases, which would lead to more pollution in the environment. Despite this concern, bismuth-based photocatalysts are frequently prepared using the solid-phase reaction approach. Consequently, well-designed bismuth-based photocatalysts can be developed by carefully controlling the operating temperature and molar ratio. The solid-phase reaction approach can be applied, as demonstrated by the successful preparation of the oxychloride compound Bi_3_O_4_Cl through the mixing of powders of Bi_2_O_3_ and BiOCl (Minkiewicz et al., 2023). The remarkable photocatalytic activity of Bi_3_O_4_Cl was attributed to its conduction band and the hybrid states at the valence band, along with the internal electric field generated between the [Bi_3_O_4_] and [Cl] layers. Subsequently, Bi_4_NbO_8_Cl was also successfully synthesized using the same solid-state reaction method (Xu et al., 2020).

## 4 Bismuth oxyhalide heterojunctions

Using UV light to photodegrade methyl orange (MO), BiOCl showed remarkable photocatalytic performance, significantly superior to commercial P25 (Zhang et al., 2006). Since then, morphological modulation, semiconductor hybridization, defect management, and face exposure adjustment for BiOX photocatalysts have attracted a lot of attention. Because of synergistic effects, the highly efficient BiOX photocatalysts often feature binary or ternary hybridized heterostructures. Semiconductor/BiOX hybrids have attracted the most research interest (Chai et al., 2009; Shamaila et al., 2011; Zhang L. et al., 2009).

### 4.1 Composition and structure of bismuth oxyhalide heterojunctions

**FIGURE 9:**
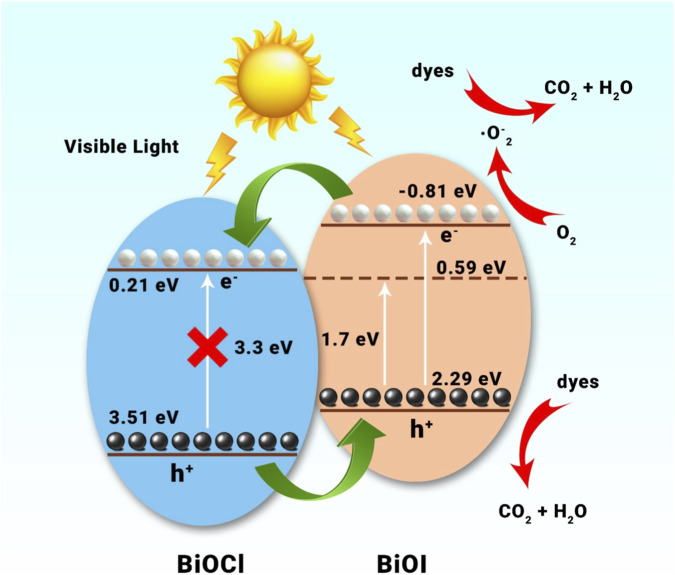
Schematic illustration of the photocatalytic reaction process over the BiOI/BiOCl heterostructure (Yang et al., 2016).

**FIGURE 10:**
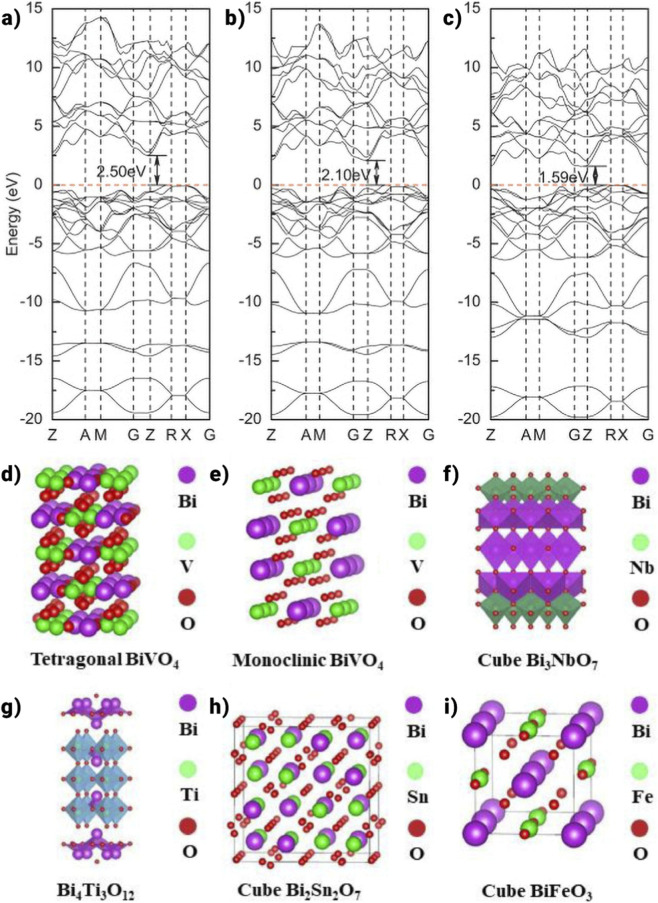
The band structures of **(a)** BiOCl, **(b)** BiOBr, and **(c)** BiOI (Zhao et al., 2012). **(d–i)** represents the crystal structure model of various bismuth-based materials (Ganose et al., 2016).

Recently, there has been growing interest in designing and developing BiOI/BiOX (Figures 10a–c) (X = Cl, Br) composite materials (Chai et al., 2009; Shamaila et al., 2011; Zhang et al., 2006; Zhang L. et al., 2009). Pure BiOI, with its narrow band gap, efficiently absorbs visible light. However, the rapid recombination of photogenerated electron-hole pairs results in low quantum efficiency, limiting its practical use in solar energy applications (Wu et al., 2020). BiOI/BiOBr composites synthesized through a one-pot solvothermal process have been reported to exhibit enhanced photocatalytic bacteriostatic activity under visible light irradiation. Yang et al. developed BiOI/BiOCl composites using a simple ultrasonic method assisted by an ionic liquid. The composites that were produced with 40% BiOI had the strongest photocatalytic activity when it comes to breaking down dye pollutants like tetracycline and rhodamine-B (Yang et al., 2016). A typical type-II charge transfer scheme is followed by the photo-excited electrons/holes due to the development of the BiOI/BiOCl heterojunction. This BiOI/BiOCl heterostructure’s photocatalytic degradation efficiency has improved dramatically as a result of the electron-hole pairs’ efficient separation and utilization. Figure 9 depicts the mechanism of the photocatalytic reaction as well as the schematic diagram of the synthesis process. Wu et al. (Lee et al., 2018) reported different findings for as-synthesized BiOX and their BiOAxB_1-x_ composites produced via a microwave-assisted solvothermal technique. BiOI demonstrated the highest photocatalytic H_2_ evolution activity among the materials due to its narrow band gap and suitably negative conduction band, facilitating water reduction under visible light. In contrast, the BiOAxB_1-x_ composites exhibited relatively poor H_2_ evolution performance, likely due to their unsuitable electronic band structure and larger band gap. Additionally, BiOX hybridized with other semiconductors shows significant potential as visible light photocatalysts. According to the BiOCl/Bi_2_O_3_ heterostructure, which was created by a chemical etching technique, it showed better photocatalytic activity than P25 for the destruction of organic pollutants that were both aqueous and gaseous (Chai et al., 2009). Without needing a co-catalyst or sacrificial agent, homogeneous C-doped Bi_3_O_4_Cl nanosheets could enhance exceptional photocatalytic OER activity under visible light. Besides, WO_3_/BiOCl (Shamaila et al., 2011), NaBiO_3_/BiOCl (Chang et al., 2010), BiOCl/BiNbO_4_/TiO_2_ (Gan et al., 2012), and Bi_12_O_17_Cl_2_/MoS_2_ (Li et al., 2016) have been fabricated and proven to show remarkable photocatalytic activities driven by visible light.

Building a Z-scheme heterojunction is one effective strategy to enhance light absorption and facilitate the transfer of light-induced electron-hole pairs alongside type-II charge transfer schemes (Jiang et al., 2019; Tang et al., 2020; Zhang X. et al., 2020). For example, using a step-by-step deposition method, a ternary Z-scheme heterostructure of BiOX (Cl, Br) - Au - CdS was developed, resulting in significantly improved photodegradation efficiency and light stability (Zhang M. et al., 2020). Another effective strategy for tuning band gap energies and enhancing overall photocatalytic activity is morphological modulation via various synthesis methods. Nano-sheets, 2D ultrathin nanoplates, and porous or hollow three-dimensional (3D) microsphere hierarchical BiOX nanostructures formed from 2D nanoplates have garnered significant attention compared to one-dimensional (1D) nanofibers (Di et al., 2017; Zhao et al., 2021; Zhou et al., 2020). When the thickness of the nanosheets drops to less than 5 nm 1, the surface atomic structure may change significantly. This could help photo-induced charge carriers diffuse more easily from the interior to the surface, preventing electron-hole pair recombination (Wu et al., 2020). The surface atomic structure may change significantly. This could facilitate the easier diffusion of photo-induced charge carriers from the interior to the surface, reducing the recombination of electron-hole pairs. Additionally, significant changes in the surface atomic structure may occur. The ultrathin layer exposes more active sites and inner atoms, enhancing light harvesting and photocatalytic efficiency. Besides hydro/solvothermal methods and chemical vapor deposition, liquid exfoliation is commonly employed to produce ultrathin nanostructures (Ding et al., 2017; Wang et al., 2020; Wu et al., 2020).

Easy and eco-friendly methods for producing uniform ultrathin nanosheets are highly sought after for practical large-scale applications (Wang et al., 2020). For instance, ultrathin 2D BiOX nanosheets (thickness less than 3 nm) with exposed {001} facets were successfully synthesized using a colloidal two-phase technique. These nanosheets showed a substantially improved activity for photocatalytic degradation of organic molecules as well as for O_2_ evolution from water splitting (Hashmi et al., 2025). A straightforward one-pot solvothermal method was used to create 3D BiOI/BiOX(X = Cl or Br) flower-like microspheres with a high specific surface area and superior visible light sensitivity (Liu et al., 2017). These microspheres showed improved O_2_ evolution performance from water splitting and photocatalytic activity towards dye degradation. Two main and straightforward techniques for creating 3D hierarchical BiOX nanostructures with high photocatalytic efficiency are hydrothermal and solvothermal procedures. Ethylene glycol (EG) is typically used as a soft template to encourage the primary 2D nanoplates to self-assemble into flower-like microspheres. Furthermore, the production of 3D porous or hollow microspheres will be aided by an ionic liquid (IL) such as 1-butyl-3-methylimidazolium iodine ([Bmim]), which functions as the iodine source, solvent, and template all at once (Di et al., 2014). The process of developing 3D sphere-like BiOBr microspheres using sodium dodecyl sulfate (SDS) in a straightforward solvothermal method can be attributed to self-assembly and inside-out Ostwald ripening growth. The microspheres have self-assembled nanoflakes of thickness 25 nm and are of diameter 2–4 μm (Zhao et al., 2016). In contrast, without the use of SDS, 3D flower-like BiOBr was also produced. The micro-flower structure, consisting of several square slices with a thickness of 160 nm, has a diameter of approximately 3–5 μm. Regarding the degradation of RhB, the 3D sphere-shaped BiOBr demonstrated more visible-light photocatalytic activity than its 3D flower-shaped counterpart. This work demonstrates that greater oxygen vacancies in 3D BiOBr microspheres, thinner nanoplates for self-assembly, and malleable nanocrystals may result in shorter diffusion channels and increased active site exposure for charge transfer and separation. Overall, morphological engineering might be used to improve 3D BiOX’s photocatalytic activity by increasing its capacity to absorb light, the number of reactive centers, and shortening diffusion paths, which would allow photogenerated charge carriers to separate and travel more quickly.

Bismuth oxyhalides, a significant category of ternary bismuth-based compounds, are characterized by exceptional Stable composition and safe, non-hazardous characteristics. The structural resemblance to Bi_2_WO_6_ is notable, with a layered lattice formed by alternating [Bi_2_O_2_]^2+^ plates and double halogen plates. These layers are held together by weak van der Waals forces, while atoms within each layer are bonded through chemical interactions. The unique layered structure, depicted in Figures 10d–f, offers sufficient room for individual atoms and orbitals, promoting polarization and generating an internal electrostatic field perpendicular to the crystal plane, which supports efficient charge separation. Various bismuth oxyhalides display different band gap widths; for example, BiOCl has a band gap of around 3.3 eV, BiOBr measures 2.7 eV, and BiOI is about 1.8 eV (Ren et al., 2020). Density functional theory (DFT) calculations reveal that the conduction band (CB) of BiOX primarily consists of Bi 6p orbitals, while the valence band (VB) is mainly composed of hybridized O 2p orbitals and X np orbitals (where n = 3, 4, and 5 for X = Cl, Br, and I, respectively) (Ren et al., 2020). Due to increased surface area, photo-induced charge carrier migration is enhanced when layered materials are reduced to monolayers or ultrathin nanosheets. In photocatalysis, the photoresponsivity of wide band gap BiOCl is predominantly in the ultraviolet region, whereas the narrow band gap of BiOI leads to rapid recombination of photogenerated electron-hole pairs, impeding practical applications. Ye et al. Ye et al. (2016b) showed that utilizing an ultra-thin layer and a bismuth-rich methodology can significantly augment the photocatalytic activity of bismuth oxyhalides (Xu et al., 2020). Utilizing nanosheets, they created ultrathin Bi_4_O_5_Br_2_ microspheres with more catalytic activity than BiOBr (2.46 μmol⋅g^−1^⋅h^−1^). Interestingly, ultra-thin BiOBr selectively converts CO_2_ into CH_4_, whereas a bismuth-rich method enhances CO_2_ to CO conversion. The catalyst surface’s ability to activate CO_2_ and H_2_O molecule interactions is greatly aided by surface oxygen vacancies (OVs). According to research by Ren et al. on hydrothermal- and chemically precipitated BiOX (X = Cl, Br, I), BiOBr demonstrated superior photocatalytic activity for CO_2_ photoreduction, with CO and CH_4_ evolution rates of 21.6 μmol⋅g^-1^⋅h^-1^ and 1.2 μmol⋅g^-1^⋅h^-1^, respectively (Xu et al., 2020). This enhanced performance was attributed to the modern band structure, oxygen vacancies (OVs), ionic radius, atomic number, and electronic density of Br. Besides the Bi-based materials discussed earlier, other Bi-containing compounds have also been reported for CO_2_ photoreduction. As shown in Figure 10g, Yu et al. synthesized high-efficiency SrBi_2_Nb_2_O_9_ nanosheets through polarization and calcination. This enhanced performance was attributed to the modern band structures, oxygen vacancies (OVs), ionic radius, atomic number, and electronic density of Br. Additionally, Figure 10h illustrates the crystal structure model of Bi_4_NbO_8_Cl (Ma et al., 2021). Figure 10i illustrates the crystal structure model of BiFeO_3_ (Ganose et al., 2016).

## 5 Optimization of bismuth oxyhalide heterojunctions and charge separation effect at the interface

### 5.1 Thickness tailoring

**FIGURE 11:**
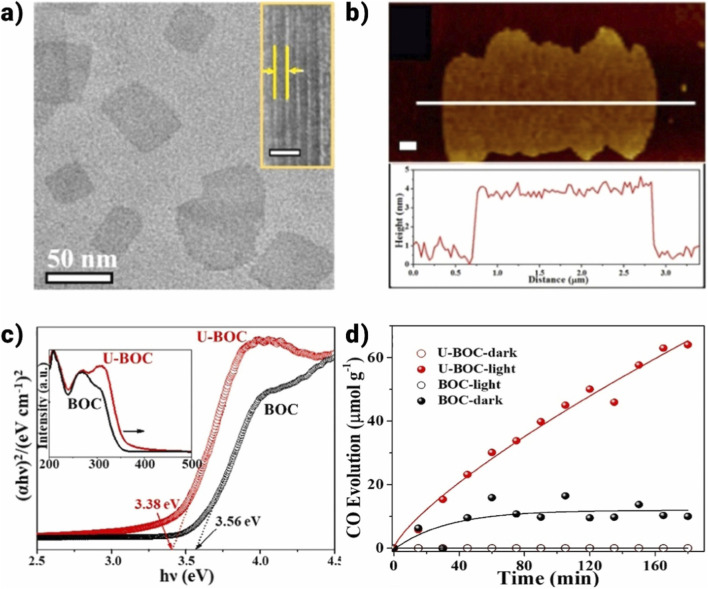
**(a)** TEM, **(b)** AFM and analogous profile, **(c)** DRS spectra, and **(d)** the CO production over ultrathin BiOCl nanosheets as a function of time (Dolla et al., 2025).

Polymerase chain reaction (PCR) performance and carrier kinetics can be significantly improved by tailoring material thickness (Karunanathie et al., 2022; Zhang Y. et al., 2021). This is explained by the benefits of atomic-level thickness reduction, including quick bulk diffusion to the surface and quicker electron and hole separation. All these elements work together to make PCR redox reactions easier. Zhang et al. provided evidence for this idea by using a solvothermal synthetic method to create ultrathin BiOCl nanosheets (U-BOC) (Chen et al., 2023). TEM and AFM images (Figures 11a,b) show the ultrathin thickness of U-BOC, while diffuse reflectance spectra (DRS, Figure 11c) indicate the wide light-harvesting range. Subsequent research showed that decreasing the thickness allows Cl atoms, as opposed to the normally exposed O atoms, to end on the exposed facet (001). In the absence of light, U-BOC’s lattice phase consists of multi-valence states of Bi, O, and Cl atoms, characterized by shorter Bi-O and Cl-O bonds due to the outward migration of Cl atoms. Upon exposure to excitation, Cl atoms migrate back into the lattice, leading to the elongation of Bi-O and Bi-Cl bonds and the normalization of Bi, O, and Cl valence states. These intrinsic changes enhance carbon monoxide (CO) production to 21.4 µmol⋅g^-1^⋅h^-1^, approximately seven times higher than that of bulk BiOCl, as shown in Figure 11d. Liquid-exfoliated atomically-thin BiOCl nanosheets (Wang H. et al., 2009), thickness-tunable BiOCl nanosheets (Hezam et al., 2018), ultrathin BiOBr nanosheets, BiOBr atomic layers, ultrathin BiOX, and BiOBr atomic layers are a few more examples that show thickness-dependent PCR activity.

### 5.2 Vacancy engineering

**FIGURE 12:**
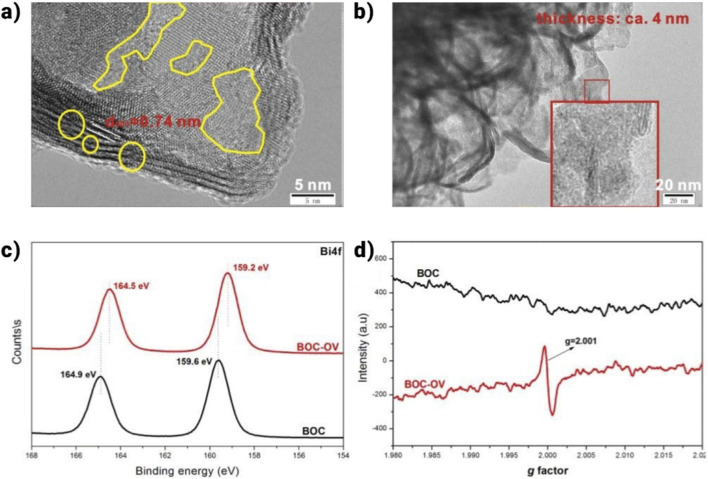
**(a)** HRTEM side-view, **(b)** TEM, **(c)** XPS spectra of Bi 4f, and **(d)** ESR spectra (Zhang L. et al., 2009).

One effective method for controlling characteristics and improving polymerase chain reaction (PCR) performance is the creation of oxygen vacancies or O_vac_. Two beneficial phenomena usually appear in BiOX after O_vac_ generation: i. the process at O_vac_ that captures photogenerated electrons and lowers the charge recombination chance, and ii. the development of faulty states, which boosts PCR and expands the range of light harvesting and efficient photocarrier production. Numerous studies have examined the benefits of O_vac_ in a variety of BiOX photocatalysts. Ma et al., for instance, used flexible BiOCl nanosheets encapsulating O_vac_, or BOC-OV, to demonstrate effective PCR. The 2D flexible BOC-OV (Figure 12a) exhibited a thickness of approximately 4 nm in the non-crystalline phase (Figure 12b). Still, numerous faults were found in its lattice structure, as shown by high-resolution X-ray photoelectron spectroscopy (Figure 12c) and electronic spin resonance (ESR) (Figure 12d). O_vac_ was confirmed by ESR. Electron-hole recombination was reduced by BOC-OV’s extended carrier lifetime and better charge separation. Redox potentials shifted because of O_vac_ production, which improved CO_2_ reduction activity and significantly raised CO generation. In addition to O_vac_, exclusive halogen or bismuth vacancies (X_vac_ or Bi_vac_) in BiOX also aid in improved PCR. For example, Bi_5_O_7_Cl, or BOC-60, was synthesized by Shi et al. at a comfortable 60 °C using light-induced dynamic Cl- vacancies (Cl_vac_).

The material shows active sites that encourage carrier separation and ease the adsorption, triggering, and conversion of CO_2_. In a similar vein, ultrathin Bi vac-rich BiOBr and BiOCl nanoplates showed effective PCR, highlighting the role that vacancies play in determining charge separation, band shape, and carrier concentration. Additionally, research has examined the cooperative effects of O and X vacancies (binary vacancies) in BiOX photocatalysts, which increase carrier kinetics and interfacial electron transfer (IEF) to adjust the electronic structure and lower CO_2_ activation barriers for better PCR results. The effectiveness of light absorption, the effectiveness of separating photogenerated electron-hole pairs, and the catalyst’s surface effects are all critical factors in the development of photocatalysis. Scholars have tackled obstacles in catalysts by employing diverse methodologies, such as element doping, morphology control, and face re-engineering. A summary of these techniques’ advancements in the field of photocatalysis may be found.

### 5.3 Bi-rich tactics

**FIGURE 13:**
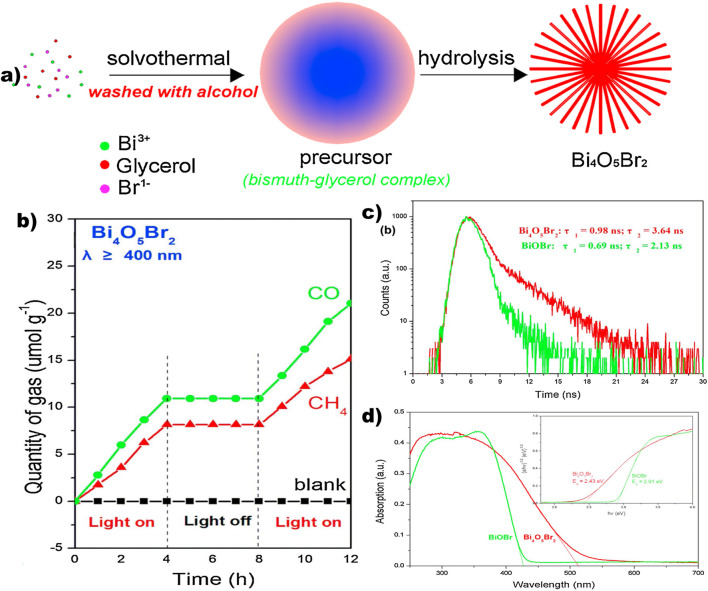
**(a)** synthesis process of Bi_4_O_5_Br_2,_ **(b)** time-dependent yield of evolved gases over Bi_4_O_5_Br_2,_ **(c)** time-resolved PL spectra, **(d)** UV-Vis diffuse absorption spectra of Bi_4_O_5_Br_2_ and BiOBr nanosheets (Ye et al., 2016b).

A vital method for optimizing polymerase chain reaction (PCR) performance and optimizing carrier kinetics is to utilize a Bi-rich strategy. This strategy aims for high-efficiency photocatalytic reactions by adjusting the conduction band (CB) potential through an increase in bismuth (Bi) content. Ye et al., for instance, produced Bi_3_O_5_Br_2_ microspheres using thin nanoplates and an atomic proportion of Bi:Br (2:1), exhibiting a strong visible light sensitivity by the solvothermal method (Ye et al., 2016b) (Figure 13a). The carrier lifespan was significantly longer at 3.64 ns after reaching a Bi-rich composition than it was for BiOBr microspheres (2.13 ns, Figure 13c). An upward shift in the CB position was caused by the increased charge separation and transfer efficiency brought about by this increase in carrier lifetime. As a result, Bi-rich Bi_3_O_5_Br_2_ microspheres produced CO in a better and more progressive manner (19.82 µmol.g^-1^.h^-1^) (Figure 13b). Additionally, Bi_4_O_5_Br_2_ (510 nm) and BiOBr (425 nm) had different absorption edges (Figure 13d). The band gap values (E_g_) for Bi_4_O_5_Br_2_ and BiOBr were 2.43 and 2.91 eV, respectively, as seen in the inset of Figure 13d. The following equation was used to assess the E_g_ values, where α,h,υ, and $E_{g}$ stand for absorption coefficient, Planck constant, light frequency, energy-independent constand and band gap.

αhυ=ahυ−Egn2 (1)

Through solvothermal techniques, Ding et al. formulated Bi-rich Bi_4_O_5_I_2_, which further contributed to the Bi-rich strategy. These Bi-rich materials showed outstanding photoreduction of CO_2_ to CO with 99.9% selectivity and a production rate of 19.82 µmol.g^-1^.h^-1^. They also had a substantially higher CB position. To sum up, the Bi-rich strategy is a crucial technique for improving carrier kinetics and improving PCR performances by adjusting CB potential through higher bismuth concentration (Ren et al., 2022).

## 6 Other bismuth-based heterojunctions

### 6.1 Titanium dioxide/bismuth-based heterojunctions

A classic example of an S-S heterojunction occurs when n-type TiO_2_ is paired with a p-type semiconductor that has a compatible energy band structure (Di et al., 2018; Zeng et al., 2014). The contact between the two semiconductors leads to the diffusion of electrons (e^−^) and holes (h^+^), resulting in the formation of a space-charge region at the p-n heterojunction interface (Ren et al., 2022). This creates a strong internal electric field that drives the photo-induced electrons (e^−^) and holes (h^+^) in opposite directions, enhancing the efficiency of charge carrier separation (Kuai et al., 2015).

**FIGURE 14:**
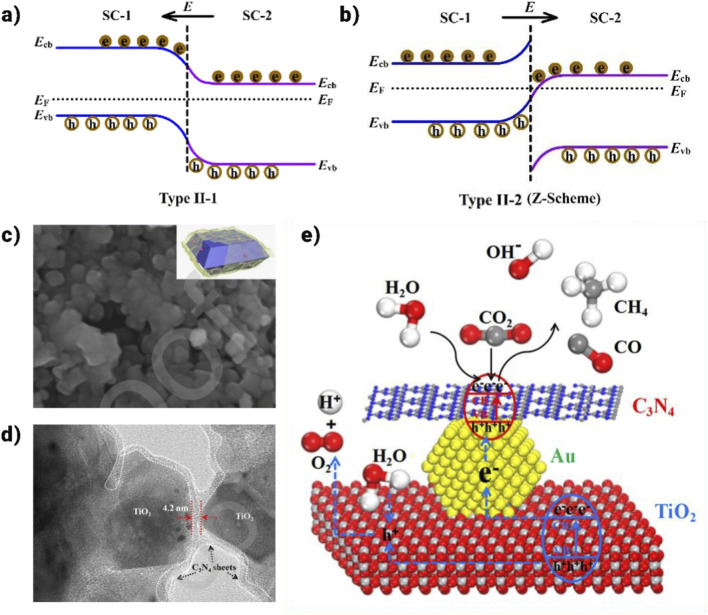
Photogenerated charge carrier transfer process for two types of non-p-n heterojunctions: **(a)** Type II-1, and **(b)** Type II-2 (direct Z-scheme), **(c)** SEM and **(d)** HRTEM images of the (Au/A-TiO_2_)@g-C_3_N_4_ catalyst, **(e)** Schematic illustrating the photocatalytic CO_2_ reduction with H_2_O to produce CH_4_ and CO over (Au/A-TiO_2_)@g-C_3_N_4_ (Ritu et al., 2018; Wei et al., 2022).

In addition to p-n heterojunctions, non-p-n heterojunctions involving TiO_2_ are also prevalent. A type II-1 heterojunction (illustrated in Figure 14a) is typically formed by two closely integrated semiconductors with staggered band structures, allowing for charge transfer at the heterointerface through band bending. In semiconductors 1 (SC-1) and 2 (SC-2), incoming light irradiation leads to the separation of electrons (e^−^) and holes (h^+^). The electron transitions from the conduction band (CB) of SC-1 to the CB of SC-2 due to the energy level difference, while the hole moves from the valence band (VB) of SC-2 to the VB of SC-1. Like p-n heterojunctions, the reverse migration of e^−^ and h^+^ in the type II-1 heterojunction enhances charge carrier separation efficiency, thereby improving the photocatalytic performance of the heterostructure system. However, the carrier transfer processes in these heterojunctions can also lead to a decrease in redox capacity, complicating efforts to achieve optimal photocatalytic activity. Recently, researchers have increasingly focused on developing all-solid-state Z-scheme heterojunctions (Van et al., 2017). In general, the band bending at the interface of direct Z-scheme heterojunctions (type II-2) facilitates the recombination of photo-induced electrons (e^−^) and holes (h^+^) with enhanced reducing and oxidizing capabilities. This configuration allows the retention of h^+^ in the more positive valence band (VB) of SC-2 and e^−^ in the more negative conduction band (CB) of SC-1 (Figure 14b). As a result, a high separation efficiency and optimal redox capability of the photo-induced charge carriers can be achieved, contributing to the strong photocatalytic performance of the Z-scheme system (Raza et al., 2020).

This section will offer a comprehensive review and discussion of recent advancements in the fabrication of TiO_2_-based all-solid-state indirect and direct Z-scheme heterojunctions, along with their applications in photocatalytic CO_2_ reduction coupled with water oxidation. This approach enhances the separation efficiency of photogenerated e^−^/h^+^ pairs and reduces ineffective charge carrier recombination, which has garnered increased attention for the photocatalytic CO_2_ reduction performance of typical TiO_2_-based all-solid-state Z-scheme heterojunctions (Wang and Tahir, 2020). This includes the construction of indirect Z-scheme systems between TiO_2_ and another semiconductor using noble metals like Pt as electron mediators. According to Tahir, Ag/TiO_2_ nano-rods and ZnFe_2_O_4_ nanospheres were physically mixed in a methanol solution while being continuously stirred to create a ZnFe_2_O_4_/Ag/TiO_2_ nanocomposite (Wang and Tahir, 2020). The enhanced connection between 0D ZnFe_2_O_4_ nanospheres and 1D TiO_2_ nanorods facilitates the transfer of photogenerated electrons and holes at the interface, compared to the point contact between 0D TiO_2_ nanoparticles and 0D ZnFe_2_O_4_ nanospheres. Furthermore, these charge carriers migrate more efficiently along the 1D nanostructure, significantly reducing the likelihood of recombination. Additionally, by recombining ineffective species within Ag nanoparticles, the UV irradiation-induced Z-scheme carrier transfer pathway ensures a high redox capability for the remaining carriers, resulting in an impressive CO production rate, which is 1,025 μmol gcat^-1^ h^-1^.

Graphitic-C_3_N_4_ (g-C_3_N_4_) is favored over ZnFe_2_O_4_ for the development of TiO_2_-based Z-scheme heterojunctions due to its ability to harness visible light fully, enhanced capacity for CO_2_ adsorption (attributed to its surface π bonds), and optimal band structure for CO_2_ photoreduction coupled with H_2_O oxidation (Rajkumar, 2020; Wu et al., 2019). Additionally, g-C_3_N_4_ can effectively capture photogenerated electrons, enhancing the efficiency of charge separation in the heterojunction. For visible-light-driven (VLD) photocatalytic CO_2_ reduction, g-C_3_N_4_ was then deposited onto the surface of Au/TiO_2_ hybrids to form a Z-scheme photocatalyst (as illustrated in Figures 14c,d) (Rajkumar, 2020). Specifically, the formation of a {001}/{101} facet heterojunction facilitates the efficient separation of photogenerated e^−^/h^+^ pairs within anatase TiO_2_. Subsequently, the photogenerated holes in the valence band (VB) of g-C_3_N_4_ recombine with the photogenerated electrons in the conduction band (CB) of TiO_2_, thereby enhancing the photoreduction of CO_2_ by the photogenerated electrons in the CB of g-C_3_N_4_ (as depicted in Figure 14e) (Rajkumar, 2020).

### 6.2 Other semiconductor/bismuth-based heterojunctions

The three types of conventional heterojunctions, type I (straddling gap), type II (staggered gap), and type III (broken gap), are characterized by their unique band configurations. In type I heterojunctions, since the valence band (VB) and conduction band (CB) of catalyst B are positioned within the forbidden band of catalyst A, photogenerated electrons and holes migrate from catalyst A to catalyst B. However, electron-hole pairs tend to collect on catalyst B, which makes it challenging for type-I heterostructures to separate them effectively. In type-II heterojunctions, catalyst A’s VB and CB sites are greater than catalyst B’s. Compared to type-I heterojunctions, type-II heterojunctions exhibit longer lifetimes for photogenerated holes and electrons. Nevertheless, the redox capacity of the two semiconductor catalysts is diminished. Because catalyst A’s CB is greater than catalyst B’s, the band gaps between these two in type-III heterojunctions are totally separated. This hinders the effective separation and transfer of photogenerated carriers between the catalysts, limiting the potential increase in photocatalytic efficiency. In summary, type-II heterojunctions offer greater efficiency compared to type-I and type-III heterojunctions. Examples of these heterojunctions include CuCo_2_S_4_/Bi_2_WO_6_ (Wang Y. et al., 2022), BiVO_4_/WO_3_ (Wang J. et al., 2022).

### 6.3 P-N heterojunction

Effective p-n heterojunction photocatalysts can be formed by combining p-type and n-type semiconductors. These p-n heterojunctions enhance electron-hole mobility by creating a self-generated electric field. Two examples of this are BiVO_4_/BiOI and BiOI/g-C_3_N_4_, which are p-n heterojunctions with excellent photocatalytic activity.

### 6.4 S-scheme heterojunction

**FIGURE 15:**
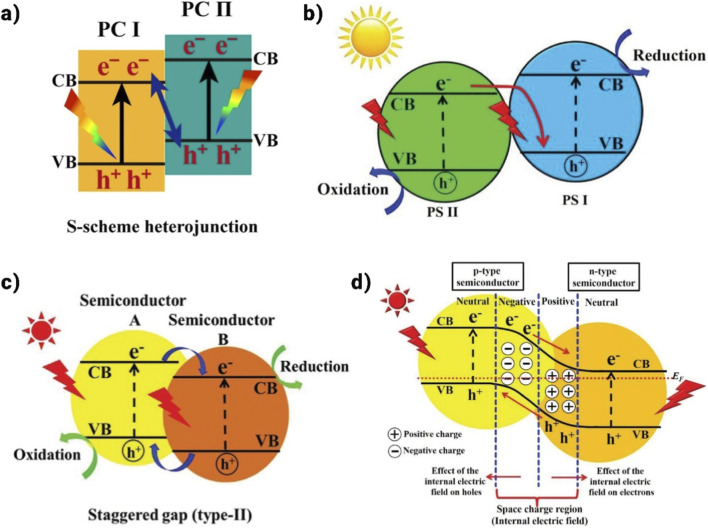
**(a)** Graphical image of S-scheme heterojunction (Fu et al., 2019), **(b)** Graphic representation of Z-scheme heterojunction (Low et al., 2017), **(c)** Graphical design of three conventional heterojunction type-II heterojunctions, and **(d)** Graphical diagram of p-n heterojunction (Low et al., 2017).

It is formed by the combination of band-staggered PC I and PC II, as seen in Figure 15a. While PC I, the oxidizing catalyst, has a bigger work function, PC II, the reducing catalyst, has a greater Fermi level. Whenever PC II electrons come closer to another semiconductor, they transfer spontaneously from PC II to PC I, where they stay till the Fermi level equalizes. This causes photocatalyst B to bend downward and photocatalyst A to bend upward. An IEF is generated at the contact to prevent further electron movement. Coulombic forces and IEF interact with the holes in the valence band (VB) of PC I and PC II to produce photo-induced reactions upon exposure to light. This maintains the CB of chemical agent PC II and the holes in the VB of chemical agent PC I, thereby retaining their high redox capability. Consequently, this approach significantly boosts the photocatalytic reaction’s efficacy. Numerous S-scheme heterostructures possessing remarkable photocatalytic activity have been produced, including Ag-CuBi_2_O_4_/CNTs/Bi_2_WO_6_, a-MnS/Bi_2_ MoO_6_, and BiOBr/Bi_2_S_3_ (Miao et al., 2022).

### 6.5 Z-scheme heterojunction

It represents a notable and innovative type of heterojunction, distinct from the conventional ones. The band structure of the Z-scheme heterojunction is illustrated in Figures 15b–d. The type-II heterojunction shares a core design principle with the Z-scheme heterojunction, namely, the use of two semiconductors with staggered gaps (Arif et al., 2019). Because semiconductor A’s Fermi level is higher than semiconductor B’s in this specific combination, semiconductor B has a lower potential. When catalysts A and B come into contact, electrons from catalyst A are transferred to catalyst B due to their differing Fermi energy levels. This process continues until the Fermi energy levels of both semiconductors reach equilibrium.

As a result of this electron transfer, electrons collect on catalyst B’s conduction band (CB) while holes grow on catalyst A’s valence band (VB). This facilitates the combination of photogenerated holes in the valence band (VB) of catalyst a with photogenerated electrons in the conduction band (CB) of catalyst B. Additionally, the intrinsic electric field created by the rearrangement of Fermi levels further promotes the recombination of the aforementioned photogenerated electron-hole pairs, which increases photocatalytic efficiency (Arif et al., 2022; Guo et al., 2024). The remarkable electron-hole separation efficiency of Z-scheme heterostructures has attracted a lot of interest. Representative Z-scheme heterostructures with exceptional performance are Bi_2_WO_6_/InVO_4_, (Li et al., 2022), CdS/BiOI (Zhou et al., 2019), Bi_2_O_3_/g-C_3_N_4_ (Wang Q. et al., 2016), and SnS_2_/Bi_2_WO_6_ (Yong et al., 2022). The different types of heterojunctions, classified by modification routes and performance metrics, are compared in Table 2.

**TABLE 2:** Heterojunctions by modification route.

Catalyst	Modification route	Target reaction	Value(s)	QE/Proxy	Stability (cycles/% retained)	Enhancement vs. reference	Ref.
Bi_2_O_2_CO_3_/BiVO_4_ (0.7:0.3)	Hydrothermal ratio-tuning	MB (vis)	33 × 10^−2^ h^−1^ (0.0055 min^−1^)	τ = 0.84 ns; AQE 6.1%	3/95%	2.4× vs. BiVO_4_	Ahmad et al. (2023), Yu et al. (2024)
Bi_2_MoO_6_-OVs/C_3_N_5_	OVs + S-scheme	TC degradation	0.089 min^−1^; 98%/75 min	AQE 8.2% @420 nm	5/92%	4.8× vs. Bi_2_MoO_6_	Feng et al. (2023)
Bi_2_MoO_6_/CdS QDs	QD S-scheme	RhB	0.0167 min^−1^; 95.8%/90 min	AQE 7.5% @450 nm	4/95%	6.2× vs. Bi_2_MoO_6_	Feng et al. (2023), Long et al. (2024)
BiVO_4_/CoPi	Co-catalyst	PEC oxidation	2.9 mA/cm^2^ @1.23 V	Lifetime ×9; AQE 9.2%	10/94%	2.9× vs. bare	Wakjira et al. (2024)
Bi_2_S_3_/BiFeO_3_ (1:3)	Composite	MG degradation	99%/60 min	AQE 7.8% @450 nm	6/90%	5.1× vs. pure BiFeO_3_	Oladipo and Mustafa (2023)
Bi_2_WO_6_/g-C_3_N_4_	S-scheme	CO_2_→CH_4_/CO	18.90/17.78 µmol g^−1^ h^−1^	AQE 0.56% @420 nm	8/93%	2.6× vs. pure	Long et al. (2024)

## 7 Applications of photocatalysts

This section highlights the latest advancements in photocatalysts for H_2_O_2_ formation/decomposition, organic synthesis, CO_2_ reduction into CO, and water splitting (Li et al., 2022), CH_4_, and other hydrocarbon fuels, photo electrocatalysis, nitrogen fixation (Shah et al., 2023), and the degradation of pollutants in both gas and liquid phases, as well as bacterial disinfection. While some of these promising applications have been reviewed individually, they focused only on a subset of constituents, such as bismuth-rich BiOX (X = Cl, Br, I), BiOCl, BiOBr, and BiOI. To date, there is no comprehensive evaluation of the wide range of SBB photocatalysts and their applications. To address this, we have compiled a summary of the diverse photocatalytic uses of SBB semiconductors, providing a clearer understanding of their photochemistry. The enhanced photoactivity of these semiconductors is primarily due to their indirect light transition and two-dimensional layered crystal structures. Photogenerated electrons are driven to the C-band through k space, which minimizes recombination charge, as evidenced by the indirect transition band gap.

### 7.1 Degradation of liquid-phase pollutants

Numerous environmental contaminants are degraded and mineralized by photocatalysis into harmless inorganic anions, CO_2_, and H_2_O. There are two categories of photodegradation on SBB photocatalysts: the elimination of gaseous pollutants and the decomposition of contaminants in water, which include organic impurities and harmful ions. The remaining electrons and holes, when exposed to photons, cause redox reactions on the photocatalyst’s surfaces. Photogenerated electrons in the conduction band (CB) of photocatalysts react with oxygen (O_2_), forming superoxide anions (O_2_ˉ) or hydroperoxide radicals (O_2_H) as shown in Equations 1–3. O_2_–also plays a role in the synthesis of OH (Equations 4, 5). Water undergoes simultaneous oxidation at the positive h^+^ (Equations 7, 8).

The oxide O_2_ photocatalysts,

$$+h\upsilon \;\to \;e^{-}+h=h^{+}$$ (3)

$$O_{2}+e^{-}\;\to \;{\cdot O}_{2}^{-}$$ (4)

$$H^{+}+{\cdot O}_{2}^{-}\;\to \;{\cdot HO}_{2}$$ (5)

$$2\cdot {HO}_{2}\;\to \;H_{2}O_{2}+O_{2}$$ (6)

$$H_{2}O_{2}+O_{2}^{-}\;\to \;{OH}^{-}+\cdot OH+O_{2}$$ (7)

$$H_{2}O+h^{+}\;\to \;\cdot OH+H^{+}$$ (8)

This section will also provide a detailed description of the photodegradation pathways of pollutants and the antibacterial and antifungal functions of SBB photocatalysts.

**FIGURE 16:**
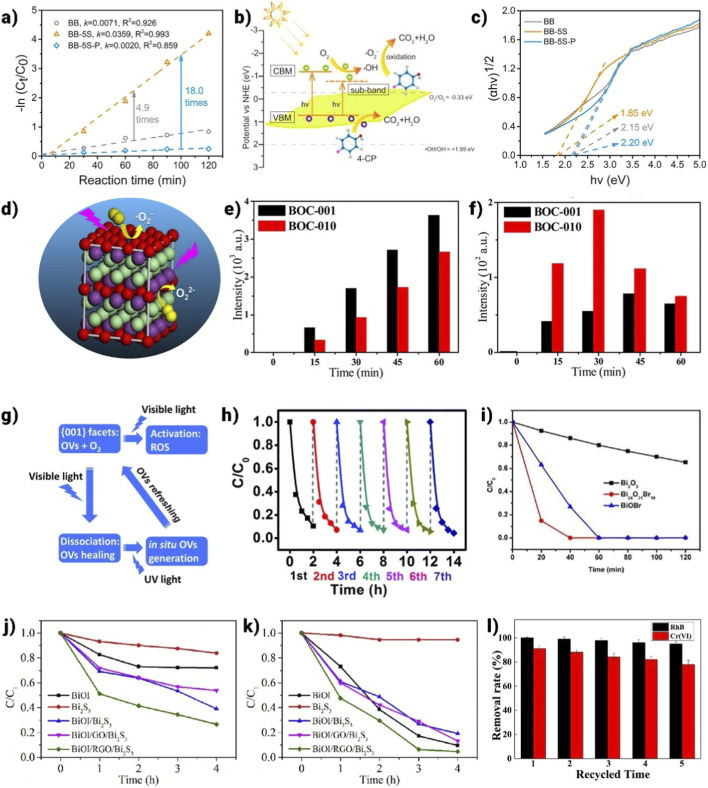
**(a)** Pseudo first-order kinetic fitting and the determined apparent rate constants (k), **(b)** Schematic illustration of the visible light photocatalytic degradation of 4-CP over Ovs-rich ultrathin BB-5S nanosheets, **(c)** The Tauc plot, **(d)** graphical abstract of surface structure-dependent oxygen activation process in BiOCl, **(e)** The amount of H_2_O_2_, and **(f)** OH from the reduction of O_2_ by photogenerated electrons over BOC-001 and BOC-010 surfaces (Zhao et al., 2013). **(g)** Photocatalytic NaPCP removal with BOC-001-3 under solar light, **(h)** *in situ* OVs refreshing on the {001} facets of BiOCl under solar light (Li et al., 2014). **(i)** Schematic diagram of the band gap of BiOBr and Bi_24_O_31_Br_10_, **(j)** Photocatalytic Cr(VI) reduction capability, **(k)** photocatalytic phenol oxidation capability (Chen et al., 2017). **(l)** Recycled removal of RhB and Cr(VI) (Gan et al., 2020).

Organic and inorganic contaminants are the two main categories used to describe wastewater contamination. The removal of contaminants in water by photocatalysis has been a widely researched topic in the early stages of photocatalysis. They can generally be broken down by photocatalysis with apparent first-order kinetics. The most common methods for describing oxidizing intermediates from the perspective of water purification are direct oxygen activation, degradation, and indirect dye sensitization process (Ahmad et al., 2023; Azhar et al., 2024; Morshedy et al., 2024; Nosaka and Nosaka, 2017; Yu et al., 2024). Furthermore, it is important to remember that environmental factors like pH and dissolved oxygen show a noteworthy influence on the rate of photocatalytic degradation. The most often utilized pollutants in the assessment of photoactivity are azo dyes. Dye molecules absorb light and get excited to produce electrons. Electrons from the dye*’s LUMO were then moved to the photocatalysts’ CB, where they were bound by molecular oxygen, resulting in the production of additional active radical species. BiOCl nanosheets with exposed (010) planes can degrade dyes via indirect photosensitization under visible light due to their large surface area and open layered structure (Chen et al., 2017). In a different investigation, carbonaceous microsphere sacrificial templates’ calcination temperatures were controlled to create BiOCl (BiOCl-HS) and Bi_24_O_31_Cl_10_ (Bi_24_O_31_Cl_10_-HS) (BiOCl-HS acquired at 400 °C, Bi_24_O_31_Cl_10_-HS obtained at 600 °C) (Jiang et al., 2012). Remarkably, the hollow architecture of the microspheres was preserved across the phase transition from BiOCl to Bi_24_O_31_Cl_10_, which turned out to be the most important component of their photocatalytic activity (Chen et al., 2017; Cui et al., 2016). When exposed to visible light, both hollow spheres had worthy RhB degradation performance, which may be attributed to both the hollow sphere structure that promoted dye adsorption and the indirect dye photosensitization procedure. Bi_24_O_31_Cl_10_-HS exhibited better visible-light-driven photoactivity than BiOCl-HS because it has more dispersive band hybrids, more efficient charge transfer, and a smaller band gap. The primary process of photodegradation is molecular oxygen activation via photocatalysis. When there is light irradiation, most dyes, colorless phenolic compounds, antibiotics, pesticides, herbicides, and other substances can be destroyed by the produced ROS, which includes O_2_, $O_{2}^{2-}$ , H_2_O_2_, and OH. A key mechanism for activating O_2_ for ROS formation is its adsorption during the molecular oxygen activation process. O_2_ typically interacts weakly with fully oxidized surfaces, but oxygen vacancies on the photocatalyst surface can provide localized electrons that easily charge the O_2_ (Yang et al., 2018). Wang et al. [1] created sulfur-doped ultrathin BiOBr nanosheets to examine the photoactivity of 4-chlorophenol. After 120 min, 98% of 4-CP was removed, which is approximately 4.9 and 18.0 times greater than that of pure BiOBr and OV-deficient S-doped BiOBr, respectively (Figure 16a). This outstanding activity might as well be attained while breaking down bisphenol analogues and sulfonamides, proving the usability of BB-xS (Yang et al., 2018). A sub-band below the conduction band, as seen in Figure 16b was created by the synergy of oxygen vacancies (OVs) and sulphur doping, enhancing visible light absorption and reducing photoinduced electron-hole recombination, according to computational and experimental results (Figure 16c). Degrading organic pollutants has also been extensively explored using the surface-dependent molecular oxygen activation procedure stated above. Zhang’s group found that on the (010) surface of BiOCl, OVs converted O_2_ to $O_{2}^{2-}$ via a two-electron transfer under UV light, while on the (001) surface, OVs converted O_2_ to $\cdot O_{2}^{-}$ via a one-electron transfer (Figure 16d). According to their findings, BOC-001 generates less H_2_O_2_ but produces more ·OH compared to BOC-010, indicating different molecular oxygen activation and photocatalytic pathways. The higher ·OH generation in BOC-001 leads to more efficient degradation of organic compounds under UV light (Figures 16e, f). OVs that were atomic-sized renewed during the dissociation process, however, can reoxidize the surface in a macroscopic perspective due to the concurrent activation and breakdown of O_2_, which blocks the O_2_ activation channel and ends O_2_ activation (Li et al., 2014). {001}-BiOCl, along with OVs, was demonstrated to efficiently and responsibly activate O_2_ for contaminant degradation under sunlight to address this issue. The {010}-BiOCl carrying OVs are easily regenerable in response to UV light Figure 16g, which helps to activate O_2_ using visible light, resulting in high efficiency and long-term stability (Figure 16h) (Li et al., 2014).

In stark contrast to earlier studies, a catalytic alloy with remarkable photo reactivity in visible wavelengths was produced by Gnayem et al., and its molecular breakdown process is unrelated to either dye photosensitization or reactive oxygen radicals. When the as-fabricated was exposed to light, BiOCl 0.875 Br 0.125 may degrade about 100% RhB and 75% acetophenone in 120 s and 180 min, respectively, with a wavelength greater than 422 nm (Gnayem et al., 2013). There was still partial degrading activity in the catalyst even after the light source was switched off. The IEF that developed between the halogen slabs and the [Bi_2_O_2_] ^2+^ positive layers, according to the authors’ hypothesis, caused effective charge separation and allowed for the operation of comparatively long-living reactive molecules that enhance photocatalytic activity in both the light-driven and dark processes. Furthermore, they postulated that these flower-like microstructures would produce longer optical paths with multiple reflections, which would enhance the photogenerated charge carriers that are available to take part in the catalytic breakdown process. Heterojunction building is another popular pollutant removal technique. With the ideal starting pH of 8, 10% BiOI/Bi_2_WO_6_ has been shown to have considerably increased photodegradation efficiency of 2,4-DCP. This increased activity was attributed to the heterojunction’s unique hierarchical structure as well as the effective charge separation and transportation it provided. Under visible light, his team manufactured a BiVO_4_/BiOCl p-n junction using a higher MO removal efficiency (Chen et al., 2015). Holes in VB were the primary cause of the MO deterioration, and dissolved O_2_ was crucial in the CB electron consumption process. Other heterojunction types also showed outstanding activity in photocatalytic degradation on a variety of contaminants, as mentioned under Sections 3.2, 3.1 (Gnayem et al., 2013; He et al., 2013). When in specific valence states, inorganic contaminants like heavy and noble metals become hazardous. Toxic metals must be transformed into benign forms or extracted entirely from wastewater for safe disposal. Using photocatalysis to change the dangerous ionic states of metals including Co(II), Hg(II), Mn(II), Ni(II), Pt(II), Cd(II), Au(II), Pb(II) Cu(II), Cr(VI), and Cr(III) is a fascinating technique (Hu et al., 2014; Li et al., 2012; Wang Q. et al., 2016). According to Shang et al., in a typical scenario, B_24_O_31_Br_10_ showed the best photoactivity among Bi_2_O_3_, BiOBr, and B_24_O_31_Br_10_ in Cr(VI) reduction, which may be eliminated in 40 min (Figure 16i) (Hu et al., 2014). They proved that the photocatalytic reduction reaction is responsible for the elevated CB level of B_24_O_31_Br_10_. Chen et al. used an electrostatic self-assembly technique to build an all SSR rGO/Bi_2_S_3_/BiOI Z-S system (Chen et al., 2017). Under visible light irradiation, the resultant photocatalysts demonstrated effective phenol and Cr(VI) elimination activities with up to 73% reduction and 95% oxidation rates (Figures 16j, k). Interestingly, their work provided a possible application strategy: using Z-scheme systems to break down organic contaminants and heavy metals simultaneously. Gan et al. observed the same outcome when they mixed BiOBr with carbon nanofibers (CCNF) generated from biomass to create a composite known as BiOBr/CCNF (Gan et al., 2020). By producing ROS, the BiOBr/CCNF photocatalysts effectively removed RhB and Cr(VI) at the same time (Figure 16l). Despite the tremendous progress made thus far, cost, efficiency, and environmental friendliness continue to be barriers to the investigation of SBB photocatalytic nanomaterials. It takes continuous effort to lower costs and increase photocatalytic performance without causing secondary pollution throughout the degradation and preparation stages. Further attention needs to be given to the photocatalyst’s reusability and the best way to recycle powdered photocatalyst.

### 7.2 Water splitting and CO_2_ reduction

Bi-based materials exhibit strong activity in photocatalytic oxidation and reduction processes, but their reduction capability can be enhanced through various modification strategies. BiOBr (001) shows considerably more advanced activity than BiOBr (010) for H_2_ production and OER, primarily due to co-exposed and highly exposed facets, which facilitate effective spatial separation of photogenerated charges (Jiang et al., 2012). The study of multilayer bismuth oxyhalides, including SrBiO_2_X, BiOX, Bi_4_NbO_8_ X, and Bi_2_GdO 4 X, for visible-light-driven water splitting is significant. The Madelung site potentials of anions encapsulate key elements of the VB structures, as indicated by experimental and DFT data. The oxide anion in the [Bi_2_O_2_] slab in Bi_4_NbO_8_Cl may play a role in the upshift of VB (Kato et al., 2017). Bi-based photocatalytic materials are shown in the literature to exhibit both significant CO_2_ photo-reduction efficacy and observable photoactivity toward water splitting.

**FIGURE 17:**
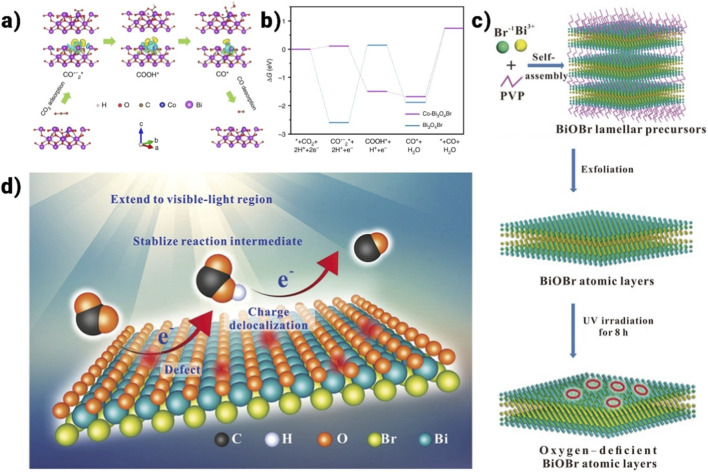
**(a)** Schematic representation of the CO_2_ photoreduction mechanism on the Co–Bi_3_O_4_Br, **(b)** free energy diagrams of CO_2_ photoreduction to CO for the Bi_3_O_4_Br and Co–Bi_3_O_4_Br, **(c)** Illustration of the formation of oxygen-deficient BiOBr atomic layers (polyvinyl propylene (PVP)), and **(d)** Advantages for CO_2_ photoreduction into CO over the oxygen-deficient BiOBr atomic layers (Cao et al., 2025; Di et al., 2019b; Niu et al., 2024).

Incorporating mono-atomic Co into two-dimensional ultrathin Bi_3_O_4_Br atomic layers (Co-Bi_3_O_4_Br) results in optimal photocatalysts with superior CO_2_ to CO selectivity, as demonstrated by the study of Di et al. DFT simulations show that single Co atoms added to Bi_3_O_4_Br can lower the activation energy barrier of CO_2_ by stabilizing COOH* intermediates and modifying the rate-limiting process to desorb CO*, resulting in a CO generation rate of 107.1 µmol g^−1^h^−1^(Figures 17a,b). BiOBr atomic layers were synthesized by Wu et al. (2018), with adequate OVs using ultra-sonication exfoliation and UV irradiation. They achieved a high conversion rate of CO_2_ to CO under visible light, with a photo-activity of 87.4 µmol g^-1^h^−1^. The integration of OVs into BiOBr atomic layers enhanced light harvesting to the visible light range. The advantages of both OVs and BiOBr atomic layers (Figure 17c) enhanced photogenerated carrier dynamics. However, charge delocalization around OVs promoted CO_2_ activation into COOH* intermediates and decreased the energy barrier of CO to increase CO yield (Figure 17d).

**FIGURE 18:**
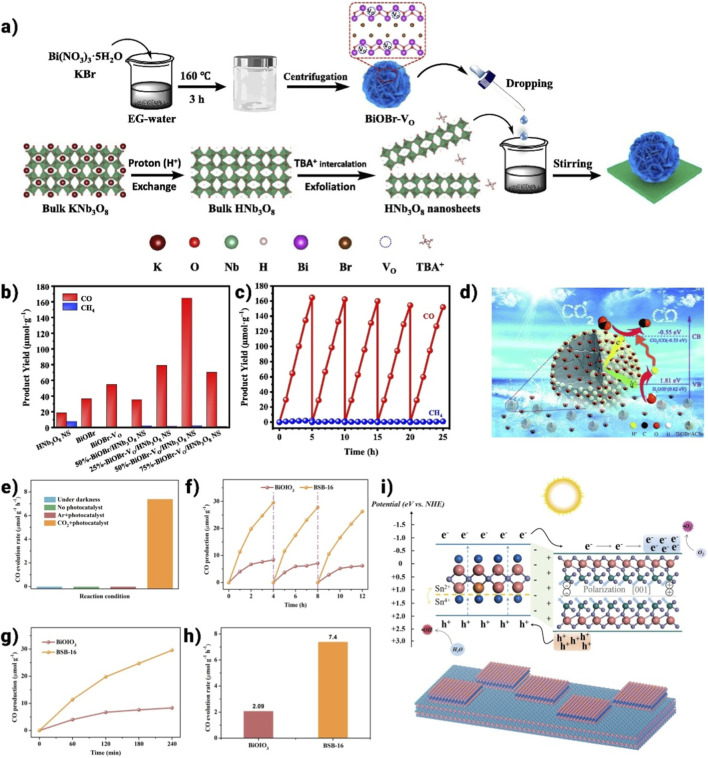
**(a)** Image of the preparatory route for BiOBr-Vo/HNb_3_O_8_ NS photocatalyst, **(b)** The results of CO and CH_4_ (5 h) in distinct samples, **(c)** Cycling performance of 50%-BiOBr-Vo/HNb_3_O_8_ NS hybrid photocatalyst (Zhou et al., 2021). **(d)** Probable technique of the photocatalytic CO_2_ reduction to CO over BiOBr/ACSs (Liu et al., 2019). **(e)** Time dependence and **(f)** noticeable rate constants for CO evolution from the photoreduction of CO_2_ over BiOBr and BSB-16 under simulated sunlight, **(g)** Controlled experiments were conducted under various conditions, **(h)** Cycling curves for CO production were recorded for the BiOBr and BSB-16 samples, **(i)** Graphic diagram of photocatalytic framework for Sn-BiOBr/BiOIO_3_ composite under light irradiation (Chen et al., 2019; Zhang et al., 2024; Zhang R. et al., 2026; Zhou et al., 2021).

The advancement of research on CO_2_ usage has been hastened by the growing respect for CO_2_ gas emissions. Given this, a viable method to minimize CO_2_ amount in the atmosphere is photocatalytic CO_2_ reduction in a few valuable fuels (Tsuji et al., 2016; Xu et al., 2018). For photocatalytically reducing CO_2_, numerous semiconductor-based photocatalyst systems have been created (Yu X. et al., 2021Yu et al., 2021a). The BiOBr-based materials have been the most extensively researched among these photocatalysts for CO_2_ photoreduction (Li et al., 2020; Wang J. et al., 2016; Yuan et al., 2017; Zhang et al., 2019). BiOBr-V o/HNb_3_O_8_NS composites were produced by Zhou et al. using self-assembly techniques (Figure 18a) (Fu et al., 2019).

The visible light absorption capacity of BiO-Br-Vo/HNb_3_O_8_ NS composites was improved upon by the combination of BiOBr-V o and HNb_3_O_8_, outperforming that of pure HNb_3_O_8_NS. The HNb_3_O_8_ NS, BiOBr, and BiOBr-V o composites have band gaps of 3.46 eV, 2.91 eV, and 2.88 eV, respectively. By looking into the photocatalytic reduction of CO_2_, the photocatalytic activity of the as-prepared BiOBr-Vo/HNb_3_O_8_NS composites was studied. The CO generation rate of the BiOBr-Vo/HNb_3_O_8_NS composites under visible light for 5 h was remarkably high at 164.6 μmol⋅g^−1^ with 98.7% selectivity, as illustrated in Figure 18b. This is three times higher than that of the BiOBr-Vo (54.7 μmol⋅g^−1^) and HNb_3_O_8_NS (18.4 μmol⋅g^−1^), correspondingly. The as-prepared BiO-Br-Vo/HNb_3_O_8_NS composites showed nearly constant selectivity after five runs, indicating that the composites’ remarkable photocatalytic reduction stability could be achieved (Figure 18c). No notable diminution in the CO or CH_4_ yield was seen. The BiOBr/ACSs composites were created by Liu and colleagues using a simple impregnation technique (Zaleska-Medynska et al., 2016). The BiOBr/ACSs composites, obtained with large specific surface areas of 792.56 m^2^/g, cumulative pore volumes of 0.31 cm^3^/g, and a greater micropore ratio of 83%, were found. Furthermore, under visible light illumination, the as-prepared BiOBr/ACSs composites had higher CO selectivity for photoreduction of CO_2_ and demonstrated exceptional photocatalytic reduction performance (54.7 μ mol⋅g^−1^⋅h^−1^) in comparison to pure BiOBr (2.39 μ mol⋅g^−1^⋅h^−1^). Finally, a potential photocatalytic process for CO_2_ reduction was examined (Figure 18d). More specifically, the photo-induced electrons of BiOBr were energized and transferred from VB to its CBM, activating the absorbed CO_2_. Additionally, the photo-induced holes left in VBM by interaction with H_2_O molecules created H^+^ for the following photocatalytic reduction of CO_2_ to CO. When considering all things together, the ACSs can provide the adsorption sites required for the physical adsorption of CO_2_ gas (Equations 9, 10).

$$h^{+}+H_{2}O\;\to \;H^{+}+\frac{1}{2}O_{2}$$ (9)

$$2H^{+}+CO_{2}+2e^{-}\;\to 2CO+H_{2}O$$ (10)

Yu and colleagues created Sn-BiOBr/BiOIO_3_ heterojunction composites (BSB-16) using metal bromide to etch *in situ* BiOIO_3_. The composites demonstrated exceptional CO_2_ reduction activity, with CO production values of 29.58 μmol⋅g^−1^ after 4 h of visible light irradiation, surpassing BiOIO_3_ (8.35 μmol⋅g^−1^). The CO production rate was 7.40 μ mol⋅g^−1^ ⋅h^−1^, over 3.54 times higher than pure BiOIO_3_ (Figures 18e,f). By substituting Ar for CO_2_ gas in reaction systems, the blank experiments further demonstrated that no CO was detected in visible light in the dark, or when no photocatalysts were added (Figure 18g). It is shown that the photocatalytic CO_2_ reduction reaction produced the CO. The high stability of the BSB-16 composites was demonstrated by the minor drop in CO generation after 12 h of continuous exposure (Figure 18h). Figure 18i was used to discuss the potential photocatalytic mechanism of Sn-BiOBr/BiOIO_3_ heterojunction composites (Kuang et al., 2023; Yu H. et al., 2021). It was proposed that the improved photocatalytic CO_2_ reduction activity was caused by the existence of an interfacial electric field. Overall, a thorough summary of the latest developments in materials based on BiOBr has been made available for photocatalytic CO_2_ reduction. Transparently, the synthesis and design of BiOBr-based materials have become research hotspots with the help of material and material scientists. But before BiOBr can be effectively utilized as a capable photocatalyst to fully utilize solar light and CO_2_ for energy generation, there are significant obstacles that need to be overcome. For instance, photocatalytic CO_2_ reduction surveys primarily focus on the reaction systems’ total reduction efficiency. There is currently a deficiency in a comprehensive understanding of the photocatalytic CO_2_ reduction process on the surface of BiOBr-based materials; the associated mechanism needs to be investigated further. Furthermore, the photocatalytic CO_2_ reduction process yields a variety of products, and the reaction systems’ exceptional selectivity towards specific hydrocarbon compound fuel generation is highly desirable for advancing the subsequent separation operation. As a result, we should take full use of this chance, as well as present fresh ideas and challenges regarding BiOBr-based photocatalysts for CO_2_ reduction, to satisfy the demands of real-world applications down the road. Since bismuth-based photocatalysts have so many unique properties and applications, they hold considerable economic promise. These catalysts are useful for many industrial processes, environmental cleanup, and the conversion of solar energy. These advantages are especially provided by layered bismuth-based (LBB) materials (Sun et al., 2024; Tao et al., 2024; Tian et al., 2022; Wu et al., 2024; Zhang Y. et al., 2026).

## 8 Challenges and future perspectives

The need to reduce environmental risks and ease energy constraints has led to the emergence of bismuth-based photocatalysts as an intriguing class of materials with a wide range of uses. With an emphasis on layered bismuth-based (LBB) materials, this study offers a thorough summary of the most current developments in the production, characterization, and uses of bismuth-based photocatalysts. Because of their distinctive coated crystal arrangement, hybrid electronic band structure, and adaptable atomic coordination, LBB materials are excellent choices for solar energy conversion applications. A widespread categorization of diverse bismuth-based photocatalysts, including bismuth oxyhalides, heterojunctions based on zinc oxide and bismuth, heterojunctions based on titanium dioxide and bismuth, and other semiconductor/bismuth-based heterojunctions, is showcased. Bi(III) has a low band gap as an outcome of the hybridization of Bi 6s orbitals with O 2p orbitals, which promotes faster charge movement and improved photo absorption. Because of their distinctive layered structures, reduced bandgaps that improve charge mobility and visible light absorption, and hybrid band structures from Bi 6s–O 2p hybridization, LBB materials are exceptional. Strengths in oxyhalides (like BiOCl) and heterojunctions with ZnO or TiO2, which improve performance in solar energy conversion, are revealed by thorough classification. However, the photoactivity of LBB photocatalysts is not up to industrial standards despite its potential for uses in H_2_ creation, pollutant purification, N_2_ reduction, CO_2_ reduction, and O_2_ generation. To analyze the microstructures and characteristics of the photocatalysts, the study uses a variety of characterization techniques. The thorough explanation also emphasizes how crucial it is to have a deep comprehension of the complex relationships between crystal structure and performance to design and build effective bismuth-based photocatalysts successfully. Scalable success is based on optimizing crystal structure-performance relationships, as confirmed by ongoing characterization approaches. Despite its potential in visible light-driven reactions, bismuth-based photocatalysts have substantial practical deployment challenges. Rapid charge recombination, poor stability, and inadequate light absorption are major obstacles that limit efficiency in practical energy and environmental applications. Poor active site exposure and weak charge separation forces cause photogenerated electron-hole pairs to recombine rapidly. Under extended use, this lowers photocatalytic performance and quantum efficiency. Reactions are further slowed by interfacial kinetics, which restrict the rates of hydrogen evolution or pollutant degradation. Even with structural benefits, efficiency is limited by rapid charge recombination, insufficient active sites, and inefficient light absorption. Numerous bismuth materials deteriorate in hostile situations, including water, or when exposed to constant radiation. Long-term reusability is hampered by structural instability caused by photocorrosion and uneven adsorption-desorption. Stability under real-world settings is still weak, with photo corrosion and scaling difficulties in large-scale synthesis limiting industrial applicability. These gaps hinder LBB photocatalysts from achieving the demands for H_2_ evolution, N_2_ fixation, and O_2_ generation.

Some variations are responsive to visible light, but they still have large bandgaps (∼3 eV for BiOCl, for example), which prevents them from fully utilizing the solar spectrum. Reduction possibilities are likewise limited by low-conduction band placements. Large-scale synthesis using techniques like hydrothermal procedures presents challenges with yield and homogeneity, and bismuth precursors can be expensive. Production at high throughput is still in its infancy. Several methods for increasing photocatalytic activity are explored to overcome this restriction, including maximizing light absorption and producing many active sites.

By combining with different semiconductors, heterojunction engineering (such as Type II or S-scheme) encourages charge separation. For improved light harvesting, doping with metals like Fe lowers bandgaps and produces defects. While surface changes (such as oxygen vacancies) improve stability and scalability by sonochemical or doping-assisted techniques, nanostructuring enhances active sites. Optimized designs for industrial viability are accelerated by high-throughput screening.

It is necessary for ongoing research to improve bismuth-based photocatalysts’ photoactivity and realize their full potential in tackling the world’s environmental and energy problems, along with the possibility of their commercialization. This thorough analysis seeks to promote research toward a sustainable future and aid in the development of effective bismuth-based photocatalysts.

### 8.1 Prospective research path

1. Advanced heterostructure and defect engineering- Future efforts should focus on constructing complex heterojunction architectures and rational doping strategies to overcome rapid charge recombination and limited light utilization. Introducing oxygen vacancies, heteroatom dopants, and metal or non-metal co-catalysts can effectively tailor band structures, enhance built-in electric fields, and extend light absorption into the near-infrared region, thereby improving overall photocatalytic efficiency.
2. Data-driven material discovery and optimization- To accelerate the identification of stable and highly active layered Bi-based catalysts, machine learning combined with high-throughput computational screening should be increasingly employed. These approaches can predict optimal compositions, defect densities, and microstructural features, reducing reliance on trial-and-error experiments and enabling faster discovery of high-performance catalysts.
3. Scalable and green synthesis strategies- For practical deployment, it is essential to develop environmentally benign, cost-effective, and scalable synthesis routes. Avoiding energy-intensive chemical vapor deposition or solvothermal processes in favor of solid-state, mechanochemical, or solution-free approaches can improve reproducibility, lower production costs, and facilitate pilot-scale manufacturing.
4. *In-situ* and operando characterization- Understanding structure-activity relationships under realistic working conditions remains a critical challenge. Advanced *in situ* and operando techniques, such as time-resolved spectroscopy and *in situ* X-ray methods, should be applied to monitor charge dynamics, surface reconstruction, and active site evolution during reactions, providing mechanistic insights beyond *ex-situ* characterization.
5. Toward practical implementation and commercialization- To bridge the gap between laboratory studies and real-world applications, life-cycle assessment and techno-economic analysis should be integrated into catalyst development. Incorporating LBBMs into pilot-scale reactors for fuel production and wastewater treatment will be essential to evaluate long-term stability, scalability, and environmental impact, ultimately promoting commercialization.
